# Influence of semiconductive shielding protrusions on the electric field distribution in ±500 kV HVDC cables

**DOI:** 10.1371/journal.pone.0356685

**Published:** 2026-08-19

**Authors:** Haolun Xu, Wei Wang, Yuanli Lu, Haotian Shi, Zhongyong Zhao, Chao Tang, Gang Lyu, Liang Cao

**Affiliations:** 1 College of Engineering and Technology, Southwest University, Chongqing, China; 2 School of Electrical and Computer Engineering, The University of Sydney, Sydney, New South Wales, Australia; 3 Guiyang Bureau, EHV Power Transmission Company, China Southern Power Grid Co., Ltd., Guiyang, Guizhou, China; Zhejiang University, CHINA

## Abstract

High-voltage direct current (HVDC) cables demand a high-performance insulation system to ensure the operational reliability. Protrusions located on cable interfaces can cause electric field distortion, resulting in insulation degradation and a reduced lifespan. By using the finite element method, a 3D model was established to explore how protrusions affect the electric field within HVDC cable insulation, considering different sizes and shapes of protrusions, the activation energy of conductivity for insulation materials and the temperature difference across the insulation. The results indicate that under full load conditions, protrusions at the interface between the insulation layer and the semiconductive shielding layer significantly affect the electric field distribution, and the height of the protrusion has a greater impact than the cross-sectional shape and size. By increasing the protrusion height from 50 μm to 300 μm, an increase of electric field distortion rate from 9.78 to 102.04 was observed. Furthermore, the electric field distortion rate near the protrusions exhibits a positive correlation with the activation energy of the insulation material, specifically increasing by 0.72 for every 0.1 eV increment. These findings provide valuable insights for the design and evaluation of HVDC cable insulation.

## Introduction

As a key component of flexible direct-current (DC) power transmission, high-voltage (HV) DC cables serve as pivotal components in long-distance energy delivery, cross-sea power transmission, and sustainable energy grid connections [1,2]. The development of HVDC cables and improvements in voltage levels have imposed increasingly stringent requirements on the electrical performance of cable insulation. At present, the voltage level of extruded DC cables in operation has reached ±525 kV [3–5]. The electrical properties of main insulation are the decisive factors affecting the service life of HVDC cables. Defects including micropores, impurities and protrusions between insulation and semiconductive shielding layer can trigger localized electric field concentration, potentially leading to partial discharge and further failure [6,7].

To ensure operation safety, the maximum allowable size of micropores, impurities, and protrusions on the interface of the HVDC cable insulation and shielding layer must be strictly controlled [8–11]. Moreover, studies have shown that a smooth surface or interface improves the breakdown strength of insulation [12–14]. Recently, Li et al. investigated the mechanisms enhancing the electrical breakdown strength of cross-linked polyethylene (XLPE) for power cable insulation, emphasizing the critical impact of local electric field distributions on insulation failure [12]. The effect of protrusions in cable insulation has been extensively studied in alternating-current (AC) cables. U. H. Nilsson noted that when calculating the field enhancement of semiconductive protrusions in HV cables, Mason’s equation is invalid and can be replaced by the Ramour equation [15]. Using a 2D model of 500 kV AC cables, Junfeng Xia et al. found that among various internal and interfacial defects, the morphology of the defect itself plays a dominant role in distorting the electric field [16]. However, the AC and DC fields follow different governing equations, so the effect of protrusions on the electric field distribution may manifest completely different patterns [17]. In the 2D case, the protrusions of DC cable insulation have also been studied. Yi Yin et al. established a 2D HVDC cable model to study the influence of the morphology of the insulating-semiconductive shielding layer on the distortion of the electric field and concluded that carbon black particles can cause protrusions at the interface, resulting in distortion of the electric field. An effective strategy for mitigating electric field distortion involves increasing the size of carbon black particles while minimizing their penetration depth into the insulation [18]. J. Wang et al. analyzed the distribution characteristics of insulation interface protrusions in HVDC cables and noted that when the height is greater than 40 μm, the protrusions between the insulation layer and the semiconductive layer will cause extreme electric field distortion [19].

For extruded HVDC cables, the electric field distribution in the main insulation is highly dependent on the conductivity of the insulating material, which relies on the electric field strength and temperature. Accordingly, both the activation energy of conductivity and the operating conditions play a fundamental role in determining the electric field distribution in cables with protrusions, particularly as this parameter often varies across materials from different suppliers. However, little attention has been given to investigating the influence of protrusions on the electric field distribution in HVDC cables considering the activation energy of conductivity and cable load conditions.

Based on a comprehensive 3D finite element method (FEM) model of a ±500 kV DC cable, protrusions with different morphologies at the interface of the insulation layer and semiconductive shielding layer are systematically simulated in this work. The electric field distributions under different activation energies of XLPE and different temperature fields within the insulation caused by varying operating conditions are further compared via 3D models.

## Establishment of the ± 500 kV DC cable model

### Structural parameters of the cable and protrusion

**Fig 1:**
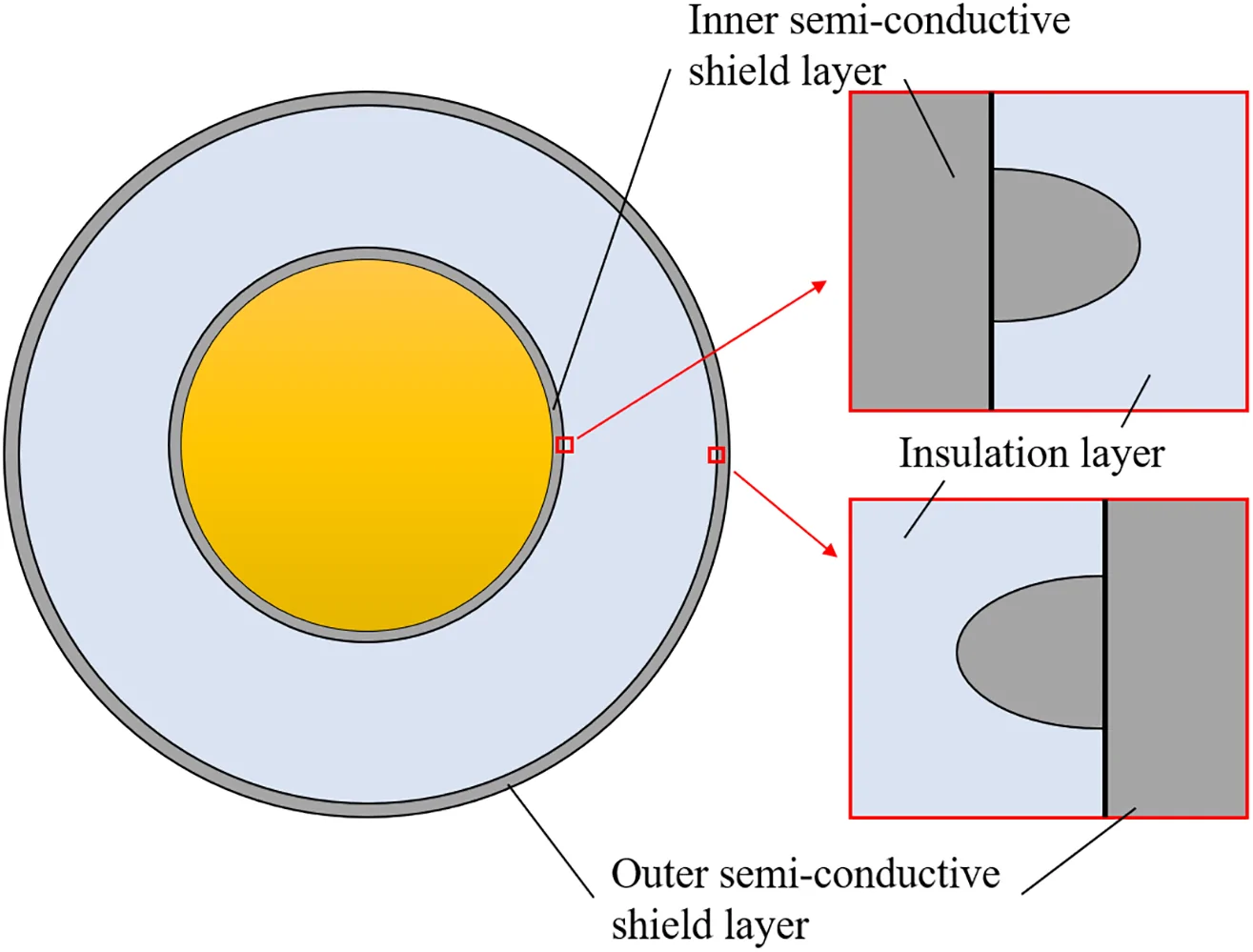
Geometry of the simplified model for a ±500 kV DC cable with a protrusion.

Typically, a ±500 kV DC cable consists of several concentric layers, including a conductor, an inner semiconductive shield, the main insulation, and an outer semiconductive shield, arranged from the innermost to the outermost layer. In this paper, a ±500 kV DC cable with an insulation thickness of 30 mm was employed to develop the simulation model. For simplification, only the four innermost layers of the cable are considered. The geometric model of the main insulation incorporating the protrusion is illustrated in Fig 1, while the corresponding structural parameters are detailed in Table 1. These geometric parameters are derived from the actual industrial manufacturing data of typical 500 kV extruded HVDC cables provided by Chongqing Taishan Cable Co., Ltd.

**Table 1:** Cable structure parameters.

Structure	Inner Diameter/mm	Outer Diameter/mm
Conductor	--	32.50
Conductor shielding	32.50	34.50
Main insulation	34.50	64.50
Insulating shielding	64.50	66.50

**Fig 2:**
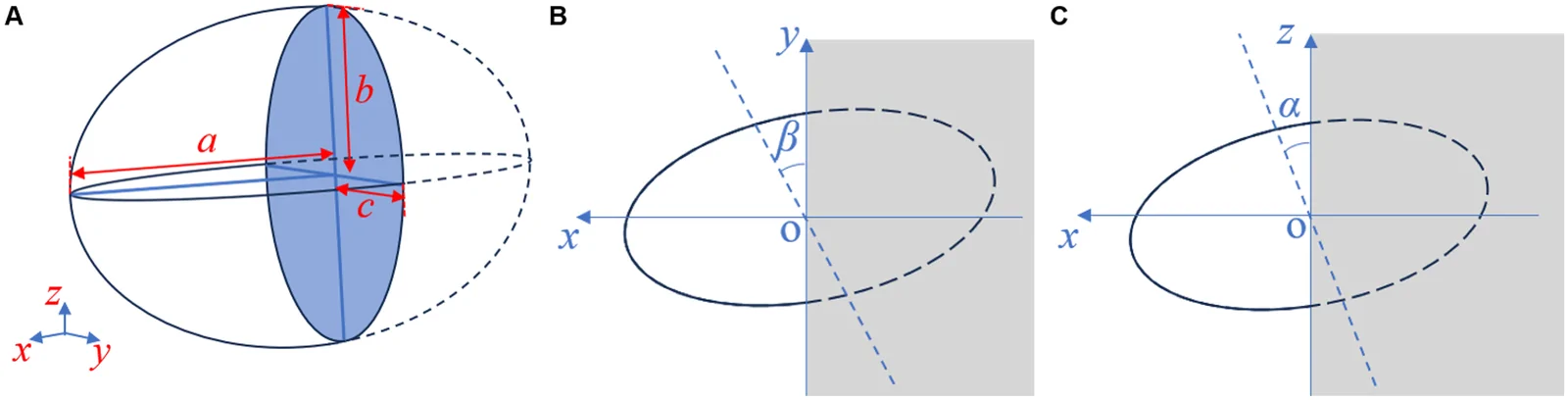
3D model of the semi-ellipsoidal protrusions. (A) 3D model of the half-ellipsoidal protrusion. (B) Protrusion *xoz* planar projection. (C) Protrusion *xoy* planar projection.

According to authoritative industrial standards for HVDC cables [8–11], high-quality manufacturing processes aim to strictly limit interfacial protrusions below 50 μm. However, agglomeration of semiconductive particles or extrusion instability during suboptimal processing can occasionally induce severe defects reaching 200–300 μm. Therefore, simulating a protrusion height range from 50 μm to 300 μm effectively covers both acceptable manufacturing tolerances and critical defect scenarios observed in industrial practice. Since these interfacial protrusions inevitably induce localized electric field enhancement, a representative geometric representation is essential for numerical evaluation. The protrusion is modeled as a semi-ellipsoid [20], as illustrated in Fig 2, to facilitate a systematic investigation into how varying morphological features impact the dielectric strength.

The semi-ellipsoidal geometry of the interfacial protrusion is characterized by three principal semi-axes: the major axis *a*, the intermediate axis *b*, and the minor axis *c*. Specifically, *a* denotes the height of the protrusion penetrating the main insulation, while *b* and *c* define its cross-sectional morphology at the interface.

Furthermore, to account for variations in orientation, the angles α and β are introduced to represent the inclinations between the intermediate axis *b* and the y axis, and the *c* axis and the z axis, respectively. By adjusting these angular parameters, the influence of protrusions entering the insulation from diverse directions can be systematically evaluated.

### Solving principles and materials

Neglecting material degradation, the governing equations for the current density within the cable under steady-state DC conditions are expressed as follows:


$$\begin{array}{c} J=\sigma E+J_{e} \end{array}$$ (1)

$$\begin{array}{c} \nabla \cdot J=Q_{j,v} \end{array}$$ (2)

where ***J*** is the conduction current density (A/m^2^), *σ* is the volume conductivity (S/m), ***E*** is the electric field intensity (V/m), and ∇ denotes the Nabla operator. In the absence of space charge, the volumetric charge source term *Q*_j,v_ is assumed to be zero.

To determine the electric field distribution, the relationship between electric potential and electric field is defined as follows:


$$\begin{array}{c} E=-\nabla U \end{array}$$ (3)

where ***E*** is the electric field, in V/m, and *U* is the electric potential, in V.

The electric field distribution within the DC cable is predominantly determined by the electrical conductivity $\sigma_{XLPE}$, which is highly sensitive to both local electric field strength and temperature. Given that the temperature within the insulation layer exhibits a radial gradient decreasing from the conductor to the outer shield, the temperature-dependent conductivity varies accordingly. This variation can be characterized by the following empirical model [21–23]:


$$\begin{array}{c} \sigma_{XLPE}=Aexp(-\frac{\varphi_{a}q_{e}}{k_{b}T})\frac{\text{sinh}(B|E|)}{\left|E\right|} \end{array}$$ (4)

where $\sigma_{XLPE}$ is DC conductivity of XLPE (S/m); *A* is a constant related to material. $\varphi_{a}$ is the activation energy of conductivity (eV). *q*_*e*_ is the elementary charge quantity (1.6 × 10^-19^ C). *k*_b_ is the Boltzmann constant (1.38 × 10^-23^ J/K). *T* is the temperature (K), and *B* is the electric field coefficient. *E* is the applied electric field (V/m).

Thermal energy within the main insulation primarily originates from conductor heat dissipation and dielectric losses. Since the heat generated by the conductor significantly outweighs the dielectric losses, the temperature field within the insulation is assumed to be dominated by the conductor’s thermal output. Under the steady-state conditions, the governing equation for heat transfer within the solid insulation is expressed as:


$$\begin{array}{c} \rho C_{p}u\cdot \nabla T+\nabla \cdot q=Q+Q_{ted} \end{array}$$ (5)

$$\begin{array}{c} q=-k\nabla T \end{array}$$ (6)

where *ρ* is the density of the main insulating shielding layer (kg/m^3^); *C*_p_ is the heat capacity under constant pressure (J/(kg·K)); *u* is the displacement vector due to material deformation (m); *q* is the heat flux vector, (W/m^2^); *k* is the thermal conductivity (W/(m/K)); *Q* is the heat density (*W*/m^3^); and *Q*_ted_ is the thermoelastic damping power (*W*/m^3^), which represents the heat generated by mechanical deformation. Since this study focuses on the steady-state operation of the cable without varying mechanical loads, the heat generated by mechanical deformation is negligible, so *Q*_ted_ equals 0.

Additionally, the relative permittivity of the main insulation is assumed to be 2.2, remaining independent of varying electric field intensities and temperature gradients. The complete set of material properties required for the simulation is summarized in Table 2 [3,24,25].

**Table 2:** The material parameters primarily used in the cable model.

Material parameters	Copper	XLPE insulation	Semiconductive shielding
Conductivity (S/m)	N/A	σ_XLPE_	0.1
Constant pressure heat capacity (J/(kg·K))	385	2200	2700
Relative permittivity	10000	2.2	1000
Density (kg/m^3^)	8940	940	1120
Thermal conductivity (W/m·K)	400	0.285	0.28

The simulated activation energy range of 0.85–1.00 eV for the main insulation was directly adopted from the research by Liu et al [24]. The materials and parameters in their study were characterized based on an actual commercial 320 kV DC XLPE cable manufactured by Zhongtian Technology Company. Utilizing the specific parameters of this commercial product ensures that the numerical model in this study accurately reflects practical industrial properties.

Since the copper conductor is metallic, its static relative permittivity is denoted as N/A. The relative permittivity value of 1000 for the semiconductive shielding layer is attributed to the carbon black loading, which alters the dielectric properties near the percolation threshold [26].

More importantly, this study focuses on steady-state DC conditions, and the electric field distribution is mathematically governed exclusively by the non-linear conductivity of the materials. Consequently, the relative permittivity primarily influences transient processes and does not participate in the steady-state potential solving equations, thus having no impact on the steady-state simulation outcomes.

For the electromagnetic-thermal coupling analysis, the ambient temperature was set at 30°C. These boundary conditions are based on the standard full-load thermal limits of XLPE DC cables. To prevent accelerated thermal aging, the maximum permissible operating temperature at the conductor is limited to 70°C [8–10]. Consequently, under typical environmental heat dissipation, the boundary temperatures at the inner and outer semiconductive shields were assigned as 70°C and 50°C, respectively, resulting in a radial temperature gradient of 20°C across the main insulation. For the electrical boundaries, the outer semiconductive shield is grounded, while a DC voltage of 500 kV is applied to the conductor.

### Model solving

Finite element method (FEM) is a robust numerical technique for solving complex partial differential equations across various disciplines including electromagnetic analysis. The core principle involves discretizing the problem domain into finite sub-regions; typically, 2D models employ triangular or rectangular elements, while 3D geometries utilize tetrahedral, prism, or pyramid elements [17]. To accurately capture the distorted electric field, the 3D model was discretized using a refined tetrahedral mesh to ensure high computational precision in this study.

Following equation (3), the local electric field at each node can be derived by calculating the gradient of the computed discrete potential distribution. To quantitatively evaluate the influence of protrusions, the electric field distortion rate is defined as the ratio of the maximum electric strength to the average electric field, expressed in following equation:


$$\begin{array}{c} \alpha =\frac{E_{max}}{E_{av}} \end{array}$$ (7)

where *E*_max_ is the maximum value of the local electric field intensity within the insulation and *E*_av_ is the average electric field strength, calculated as the ratio of the applied DC voltage to the total insulation thickness.

The 3D electromagnetic-thermal coupling model in this study was implemented and solved using commercial software ANSYS.

## Results and discussion

### Validation of the simulation model

Due to the inherent technical challenges in precisely fabricating and measuring specific-sized defects at the microscopic interface, current research on such issues relies predominantly on numerical computations. To validate the proposed simulation framework, the numerical results were compared with the analytical model developed by Gutiérrez S. Under idealized symmetric conditions, the localized electric field enhancement trends calculated by our model align closely with classical analytical laws, wherein the electric field distortion rate scales proportionally with the geometric aspect ratio of the protrusions [27–29]. It must be emphasized, however, that traditional 2D simulations are mathematically restricted to perfectly symmetric defects, which deviates significantly from real-world conditions. The 3D model established in this study is capable of quantitatively evaluating the complex impacts of highly irregular protrusions—characterized by asymmetric cross-sections and various spatial inclination angles—on localized electric field distortion within actual extruded cables. Nevertheless, as a limitation, this model simplifies the transient process of space charge accumulation.

### Electric field distribution in the cable without semiconductive shielding protrusions

**Fig 3:**
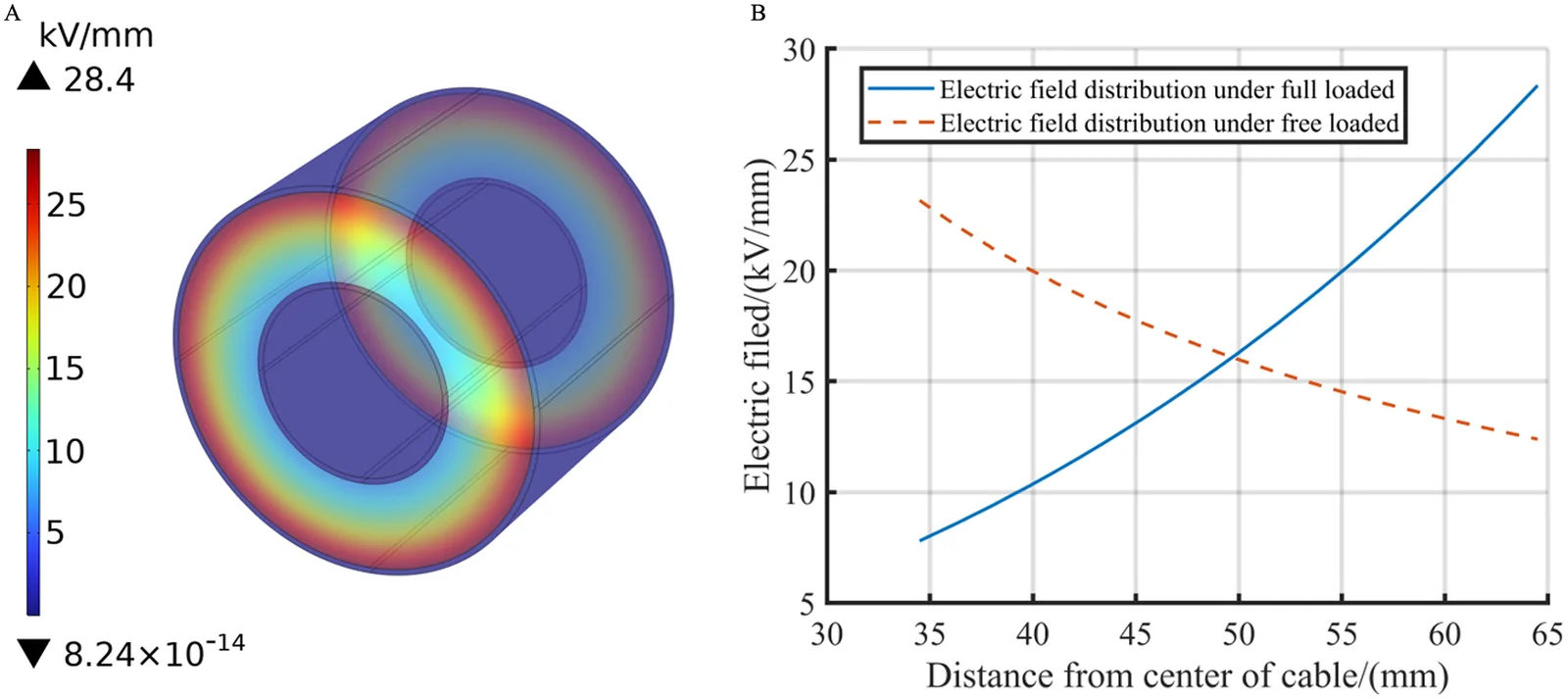
Electric field distribution in cables without semiconductive protrusions.

As illustrated in Fig 3, the radial temperature gradient induces an electric field reversal within the insulation layer, causing the distribution to change from decreasing to increasing radially from the inner to the outer interface. Under full-load condition, the minimum electric field strength |***E***|_min_ of 7.8 kV/mm is observed at the interface between insulation and inner semiconductive shield layer. Conversely, the maximum electric field strength |***E***|_max_ reaches 28.4 kV/mm at the outer interface between the insulation and semiconductive shielding,

### Comparison of electric field distribution with protrusions existing on the inner and outer interfaces of the main insulation

Protrusions are added at the interfaces of both the inner and outer semiconductive shields with the insulation layer. The semi-axis is set as *b* = *c* = 50 μm, resulting in a circular cross-sectional profile at the interface. The protrusion height *a*, representing the penetration depth from the semiconductive shield into the insulation is also specified as 50 μm.

**Fig 4:**
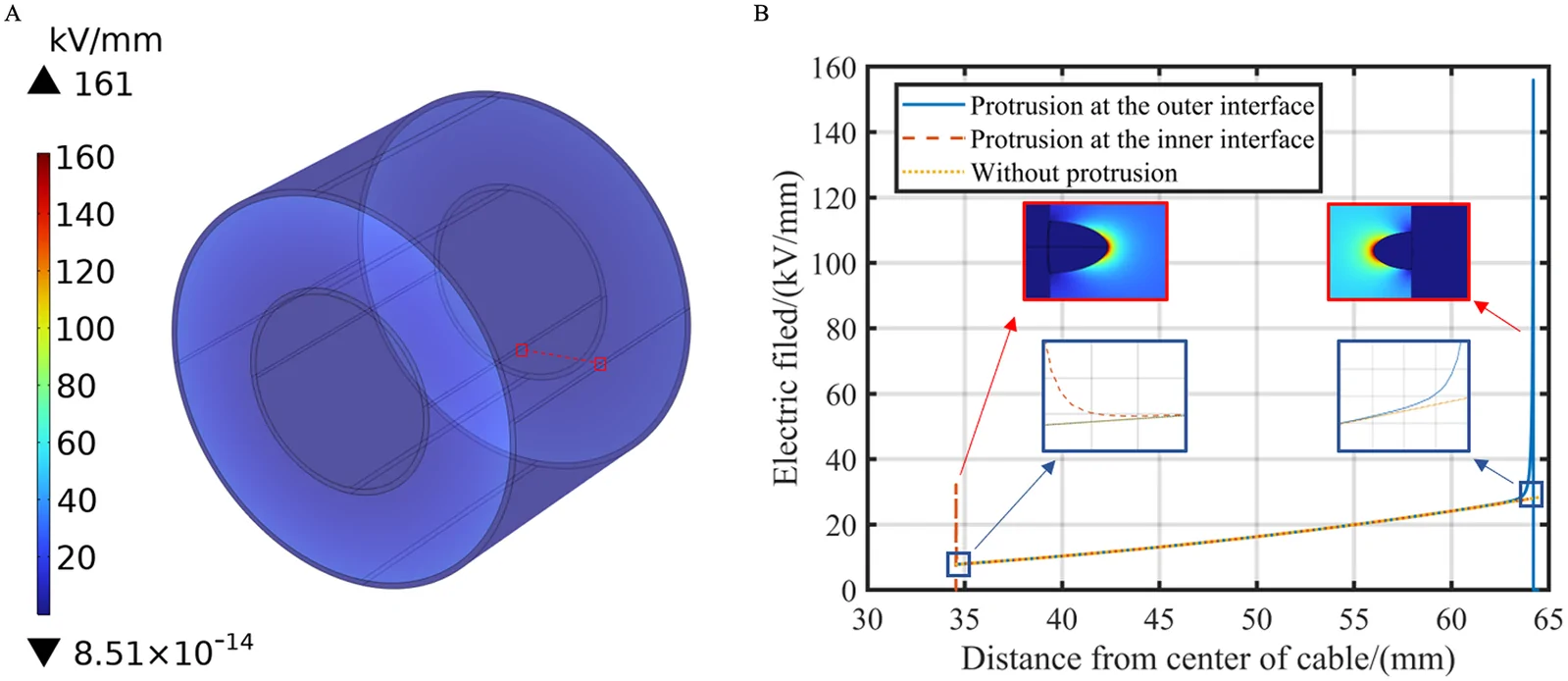
Comparison of Electric Field Distributions for Inner and Outer Interfacial Protrusions.

The electric field distortions induced by protrusions at the inner and outer semiconductive shielding interfaces were compared under full-load conditions. The simulation results of the electric field distribution for protrusions at different interfaces are shown in Fig 4. The results indicate that while these protrusions exert a negligible effect on the global electric field profile within the main insulation, they significantly amplify the peak local electric field intensity. The maximum electric field is highly concentrated at the protrusion tips, characterized by a substantial yet localized enhancement of the field magnitude.

Specifically, the peak electric field intensity near the protrusions at the inner insulation-shielding interface is 32 kV/mm. In sharp contrast, this value escalates to 161 kV/mm for protrusions located at the outer interface. These results demonstrate that under full-load conditions, electric field distortion at the outer semiconductive layer is substantially more severe than that at the inner layer.

Given that higher electric field concentrations pose a more significant threat to dielectric integrity, this study primarily focuses on investigating field distortions associated with protrusions at the outer insulation-shielding interface.

### Electric field distribution in cables with different shapes of semiconductive protrusions

**Fig 5:**
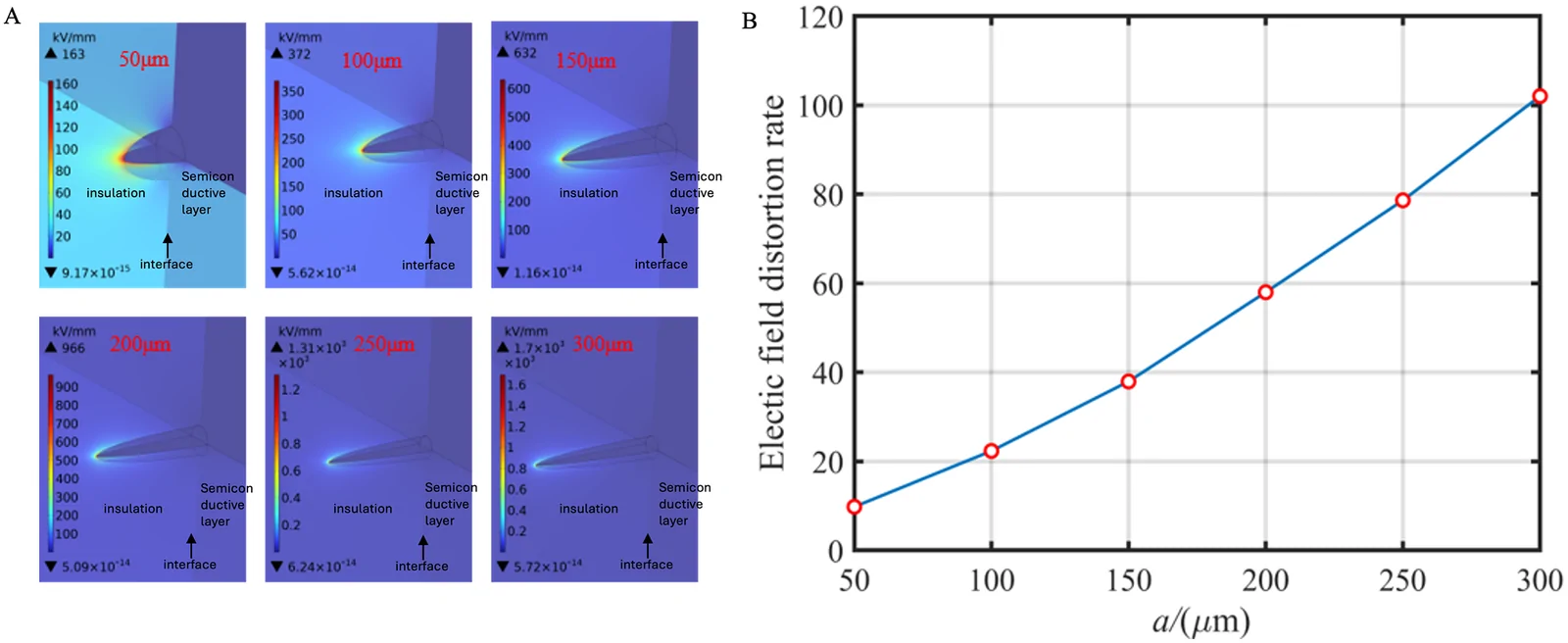
Effect of protrusion parameter *a* on the electric field distortion rate. (A) The electric field distribution near the protrusion for different values of *a.* (B) The variation in the electric field distortion rate with parameter *a.*

Due to the axisymmetric nature of the cable structure, protrusions situated at an equivalent radial distance from the cable axis exert an identical influence on the electric field distribution. To evaluate the impact of protrusion height on field distortion, the axis *a* is aligned with the cable’s radial direction. While *b* and *c* remain at 50 μm, the value of *a* is varied from 50 μm to 300 μm in increments of 50 μm. Fig 5a shows the influence of protrusion height on the electric field distribution. The maximum electric field intensity is concentrated at the protrusion tip and escalates as the height *a* increases. Specifically, the maximum electric field intensity rises from 163 kV/mm at *a* = 50 μm to 300 kV/mm when *a* = 300 μm.

The corresponding electric field distortion rates are presented in Fig 5b. The electric field distortion rate scales with the protrusion height *a*, rising from 9.78 at *a* = 50 μm to 102.04 when *a* = 300 μm. Notably, the relationship between the electric field distortion rate and the protrusion height exhibits a pronounced nonlinear trend.

To evaluate the severity of these distortion rates, a dangerous threshold is defined based on the intrinsic DC breakdown strength of commercial XLPE, which typically ranges from 100 to 500 kV/mm under operating temperatures [30]. Based on the distortion rate definition in Equation (7), the critical condition for insulation failure occurs when the maximum localized electric field $E_{bd,min}$ reaches the lower limit of the material’s breakdown strength. Under full-load conditions, the average electric field $E_{av}$ across the insulation is approximately 16.6 kV/mm. Therefore, the critical distortion-rate threshold $\alpha_{critical}$ can be mathematically calculated as:


$$\begin{array}{c} \alpha_{critical}=\frac{E_{\text{max}(critical)}}{E_{av}}=\frac{E_{bd,min}}{E_{av}}=\frac{\text{1}\text{0}\text{0}}{\text{1}\text{6}.\text{6}}\approx \text{6}.\text{0} \end{array}$$ (8)

Based on this defined threshold, the simulated distortion rate of α exceeds $\alpha_{critical}$ and is classified as dangerous, indicating a risk of insulation failure. Furthermore, an extreme distortion rate of α=102.04 implies a localized electric field exceeding 300 kV/mm, posing a risk of immediate breakdown. Beyond immediate failure risks, long-term exposure to these super-threshold fields significantly accelerates electrical treeing and aging according to the classical inverse power law of electrical aging prematurely terminating the service life of the HVDC cable [30].

**Fig 6:**
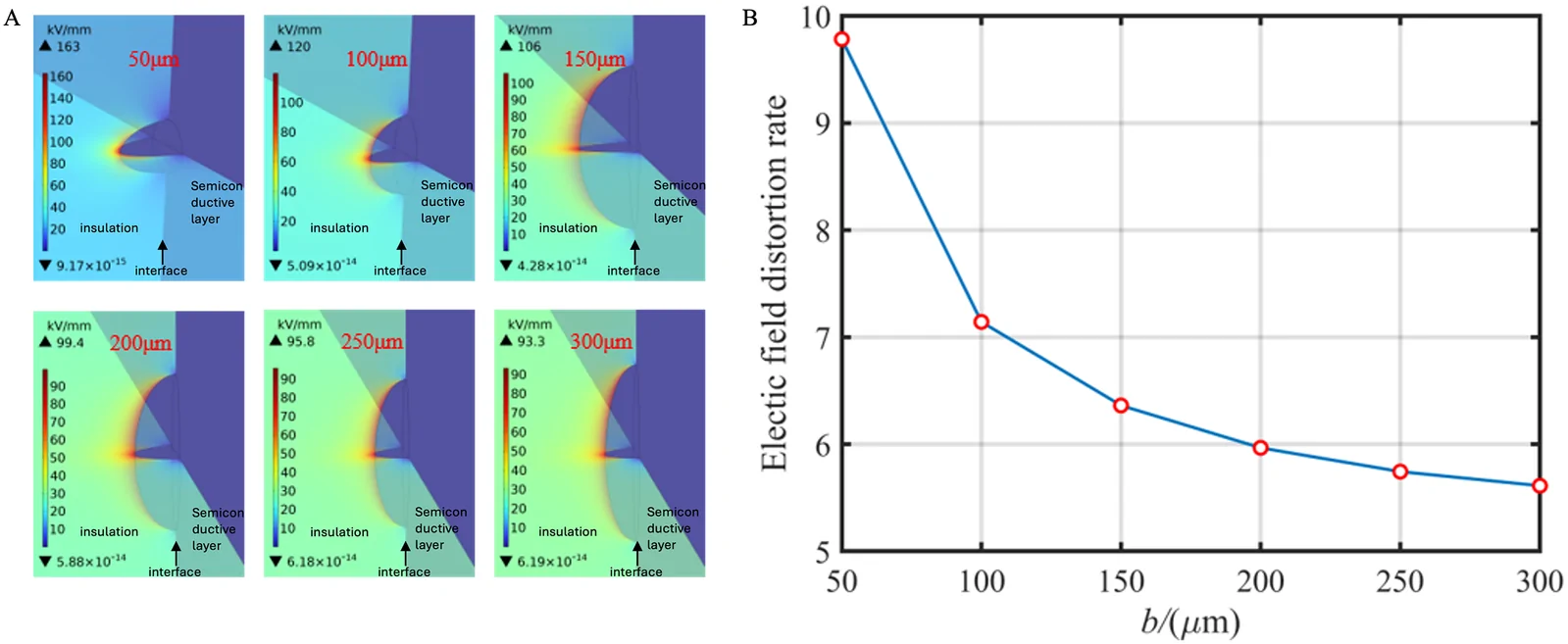
Effect of the change in the protrusion parameter *b* on the electric field distortion. (A) The electric field distribution near the protrusion for different values of *b.* (B) The variation in the electric field distortion rate with parameter *b*.

Maintaining the semi-axes *a* and *c* at 50 μm, the value of *b* is varied from 50 μm to 300 μm in increment of 50 μm. Fig 6a illustrates the influence of cross-sectional geometry of protrusion on the electric field distribution as a function of parameter *b*. In contrast to the effect of height *a*, the peak electric field intensity decreases as *b* increases; specifically, field strength drops from 163 kV/mm at *b* = 50 μm to 93.3 kV/mm when *b* = 300 μm.

Fig 6b demonstrates the electric field distortion rate diminishes as *b* increases. The distortion rate declines from a peak of 9.78 at *b* = 50 μm to 5.61 when *b* is expanded to 300 μm. Furthermore, as *b* increases, the rate of reduction in the distortion rate gradually levels off, indicating a non-linear attenuation characteristic.

**Fig 7:**
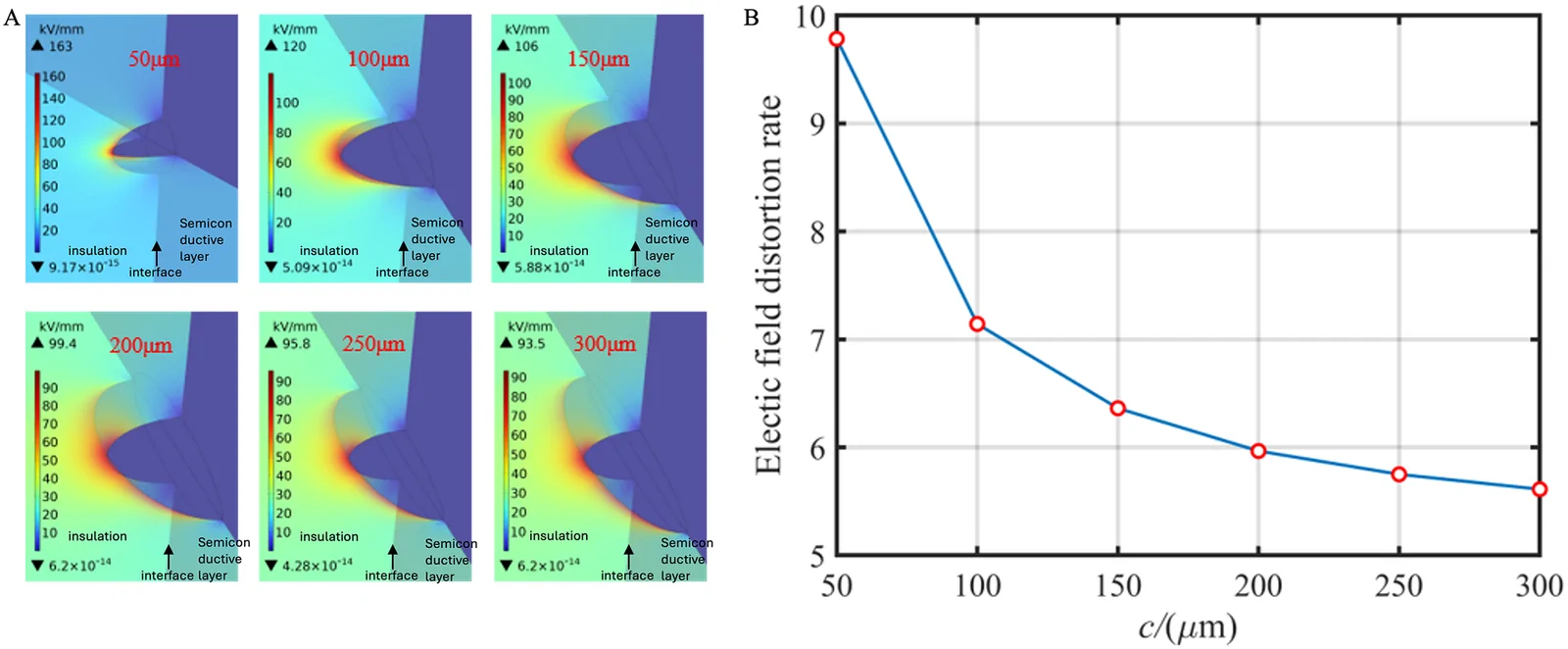
Effect of the protrusion parameter *c* on the electric field distribution and distortion rate. (A) The electric field distribution near the protrusion for different values of *c.* (B) The variation in the electric field distortion rate with parameter *c.*

While *a* and *b* remain at 50 μm, the value of *c* is varied from 50 μm to 300 μm in increments of 50 μm. Fig 7a illustrates the impact of protrusion’s cross-sectional geometry on the electric field distribution as a function of *c*. The maximum electric field intensity inversely correlates with *c*, declining from 163 kV/mm at *c* = 50 μm to 93.3 kV/mm when *c* = 300 μm.

As evidenced in Fig 7b, the electric field distortion rate diminishes as *c* increases, falling from 9.78 to 5.61. This result further confirms that expanding the horizontal dimensions of the protrusion effectively mitigates field concentration.

**Fig 8:**
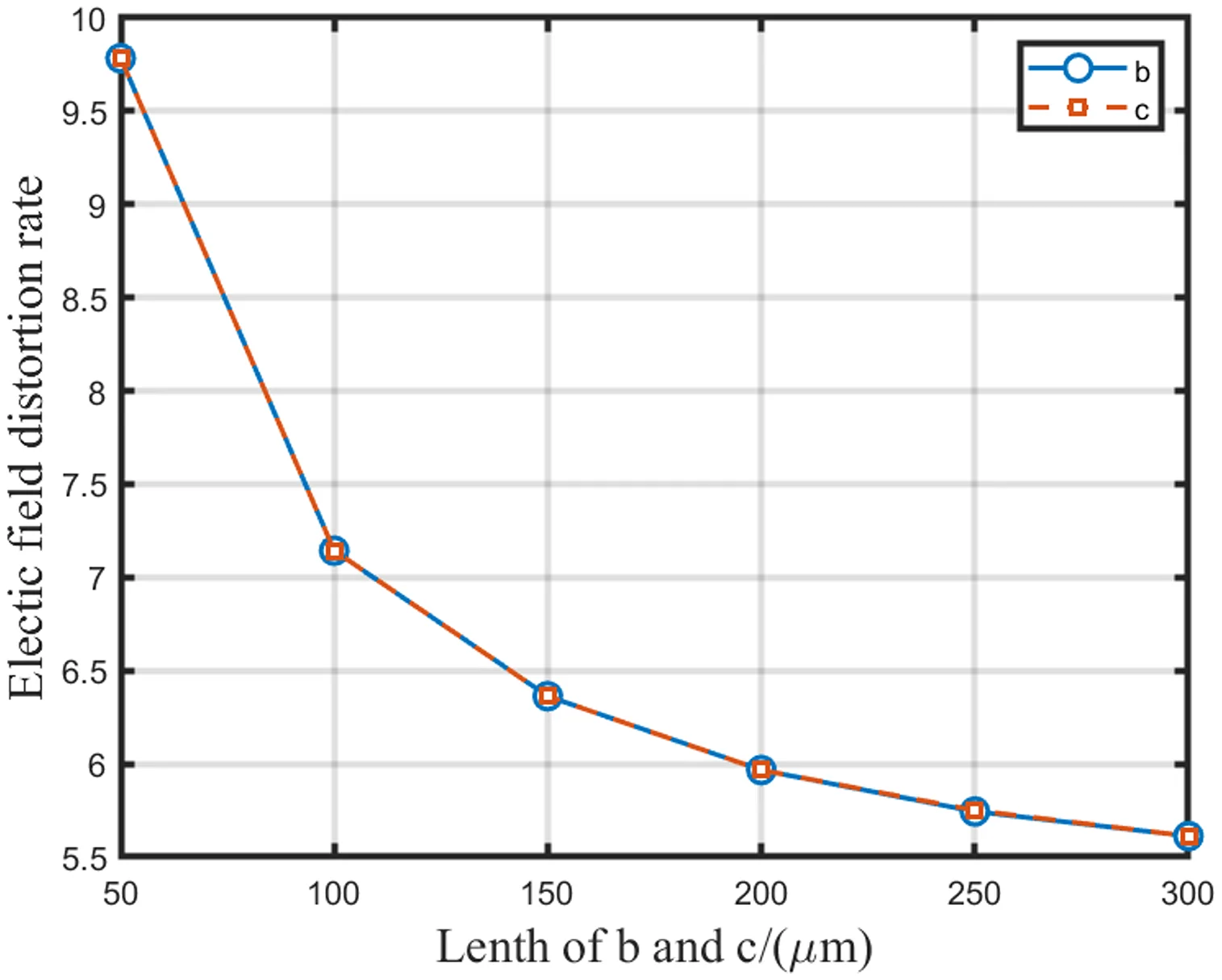
Influence of *b* and *c* on the electric field distortion rate.

To evaluate cross-sectional symmetry, the impacts of parameters *b* and *c* on field distortion were compared in Fig 8. As illustrated, increasing either parameter leads to a consistent reduction in the electric field distortion rate. This suggests that at the insulation-shielding interface, the cross-sectional dimensions exert a symmetrical influence on field concentration regardless of orientation.

**Fig 9:**
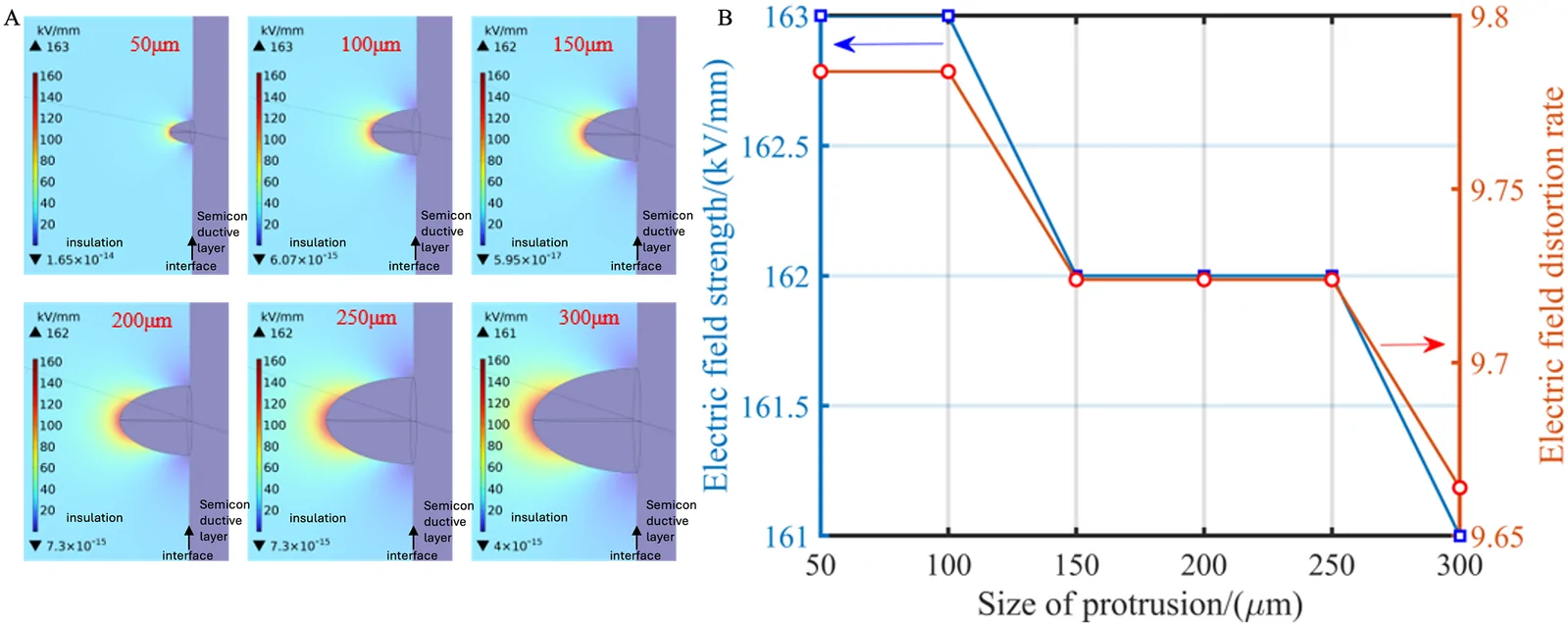
The distortion of the electric field varies with the protrusion. (A) Electric field distribution for different protrusion sizes. (B) The variation in the electric field distortion rate with the size of the protrusion.

By simultaneously increasing *a*, *b*, and *c* from 50 μm to 300 μm while maintaining a constant 1:1:1 ratio, the electric field distributions for various protrusion sizes are obtained in Fig 9a. The maximum electric field intensity slightly decreases from 163 kV/mm at *a* = *b* = *c* = 50 μm to 161 kV/mm when *a* = *b* = *c* = 300 μm.

As shown in Fig 9b, the electric field distortion rate exhibits a marginal decrease as the protrusion dimensions scale up. Specifically, the distortion rate slightly declines from 9.78 at *a* = *b* = *c* = 50 μm to 9.66 when *a* = *b* = *c* = 300 μm. Compared to the significant variations induced by changing individual *a, b* or *c* independently, the field distortion remains relatively stable when protrusion size is scaled proportionally.

**Fig 10:**
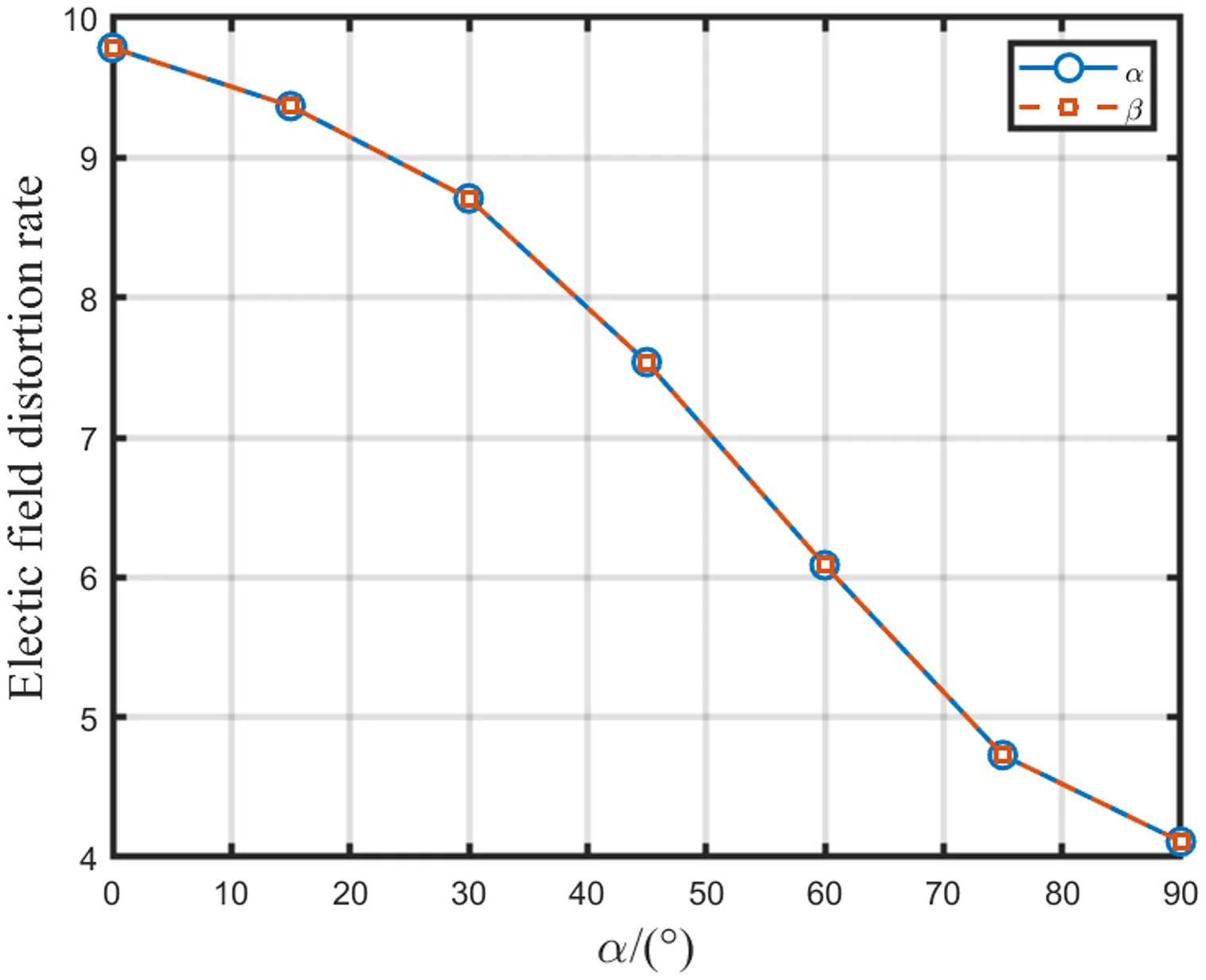
Influence of Protrusion Orientation on the Electric Field Distortion Rate.

Beyond geometry and dimensions, the orientation of the protrusion relative to the insulation layer also influences the electric field distribution. Fig 10 presents the distortion rates obtained by varying the angles *α* and *β* from 0° to 90° in 15° increments. For a fixed *β* = 0*,* the distortion rate diminished as *α* increases, dropping from 9.78 at *α* = 0° to 4.11 at *α* = 90°. Similarly, varying *β* while *α* = 0° yields an identical trend. These results indicate that for a semi-ellipsoidal protrusion of a fixed geometry, the field distortion remains invariant as long as the inclination angle relative to the cable’s radial direction is constant.

### Electric field distribution in cables with different conductivity activation energies for the main insulation

Even for identical cable types, such as ±500 kV HVDC cables, the activation energy of the main insulation may vary due to manufacturing discrepancies. As defined by equation (6), conductivity is intrinsically linked to the local electric field, temperature, and activation energy. Since the steady-state electric field distribution within the insulation is governed by this conductivity, both the temperature field and the material’s activation energy are expected to significantly influence the field distortion induced by protrusions.

Under full-load condition, the insulation layer exhibits a radial temperature gradient, decreasing from the inner to the outer shield. Since conductivity is a function of both temperature and activation energy, the specific impact of activation energy on the conductance profile and the resulting electric field distortion can be evaluated for a fixed temperature distribution.

**Fig 11:**
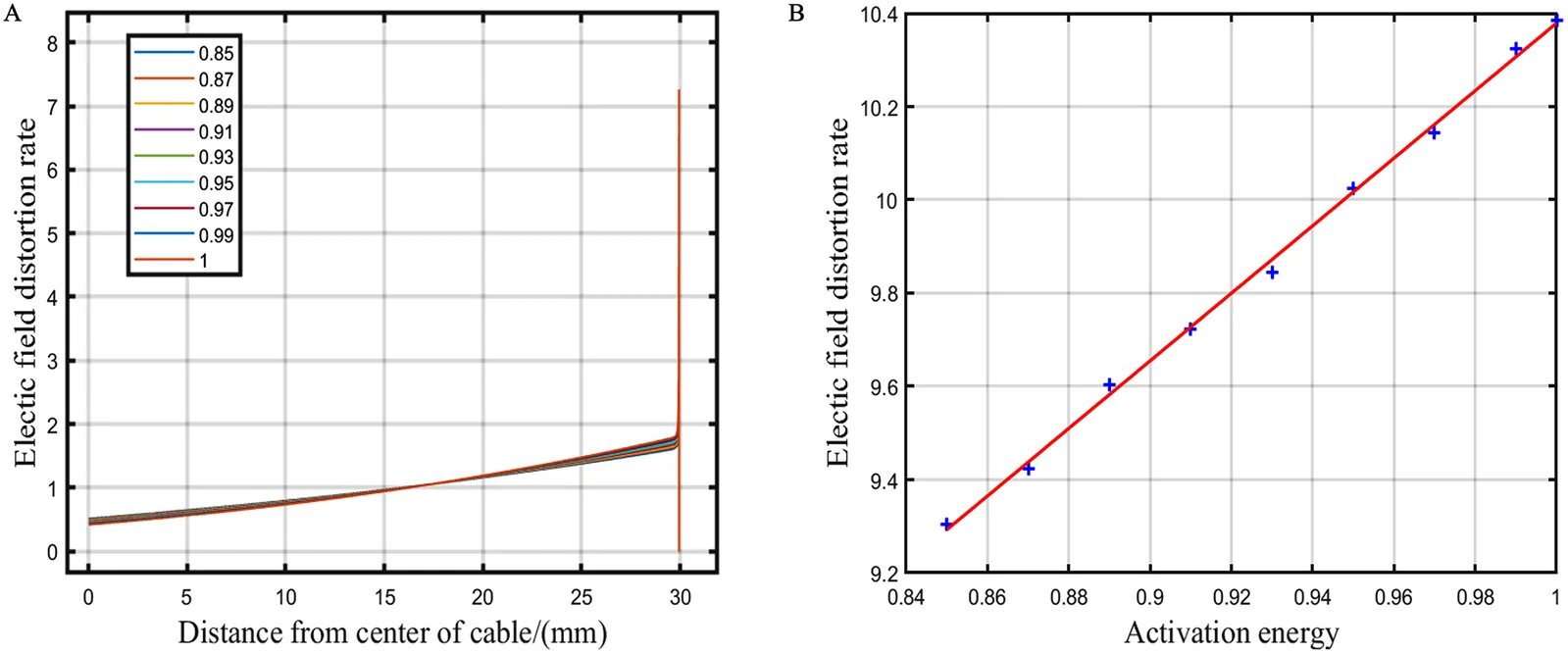
Influence of Activation Energy on Electric Field Distribution and Distortion Rate. (A) The electric field distribution in the insulation when the activation energy changes. (B) The variation in the electric field distortion rate with activation energy.

The temperatures at the inner and outer insulation-shield interfaces were set at 70°C and 50°C, respectively. Protrusion parameters *a*, *b*, and *c* were fixed at 50 μm with orientation angles *α* = *β* = 0°. The activation energy *φ*_*a*_ was varied from 0.85 eV to 1.00 eV in increments of 0.02 eV [23,31]. Fig 11a illustrates that increasing *φ*_*a*_ intensifies the electric field reversal, thereby diminishing the field intensity near the inner shield while amplifying it near the outer shield. Consequently, as shown in Fig 11b, the electric field distortion rate rises near-linearly from 9.30 at *φ*_*a*_ = 0.85eV to 10.38 at *φ*_*a*_ = 1.00eV.

The pronounced sensitivity of the distortion rate to activation energy can be explained by the fundamental conductivity mechanism of the insulation. Under high DC stress and thermal gradients, steady-state conduction in commercial XLPE is predominantly governed by ionic conduction and carrier hopping [3,25]. The activation energy represents the potential barrier that these charge carriers must overcome to migrate through the polymer matrix. Consequently, a higher activation energy makes the macroscopic conductivity highly dependent on temperature. Because the outer shield interface operates at a lower temperature due to the radial thermal gradient, the local conductivity drops much more sharply for materials with high activation energy. Since the steady-state DC electric field is distributed inversely proportional to conductivity, this severely exacerbates the electric field concentration at the colder outer protrusion.

### Electric field distribution in cables with different temperature profiles across the main insulation

**Fig 12:**
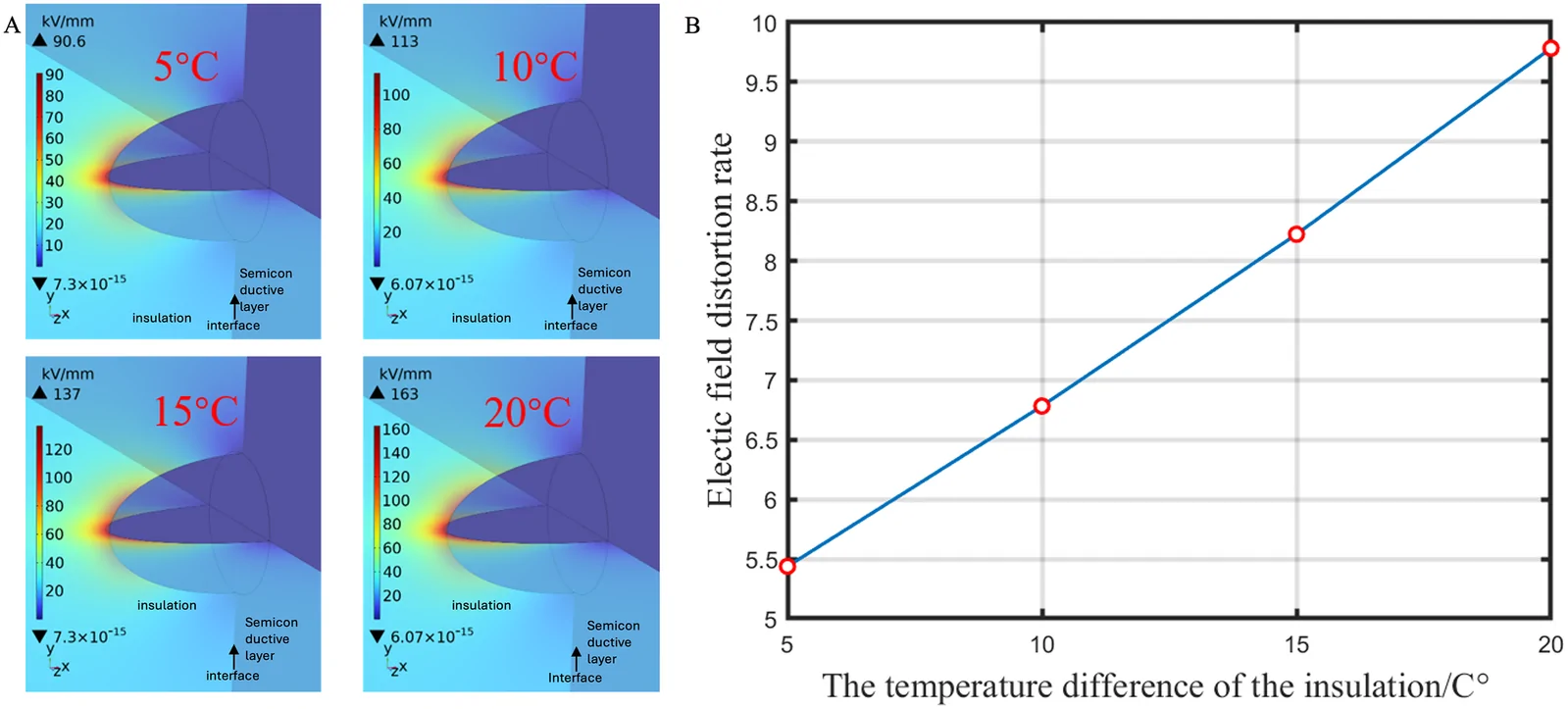
Effect of the cable operation state on the electric field distortion rate. (A) Electric field distribution under different temperature fields. (B) The variation in the electric field distortion rate with respect to the insulation temperature.

With *a*, *b* and *c* fixed at 50 μm (*α* = *β* = 0°) and the activation energy maintained at 0.91 eV, the temperature difference (ΔT) across the insulation was varied from 5°C to 20°C to simulate the transition from light-load to full-load conditions in Fig 12a. The peak electric field intensity increases significantly with ΔT, rising from 90.6 kV/mm at ΔT = 5°C to 163 kV/mm at ΔT = 20°C.

As illustrated in Fig 12b, the electric field distortion rate scales linearly with the temperature difference (ΔT) across the insulation. Specifically, the distortion rate rises from 5.48 under light-load conditions ΔT = 5°C to 9.78 under full-load conditions ΔT = 20°C.

## Conclusion

In this study, a 3D finite element model was developed to evaluate the electric field distortion induced by interfacial protrusions in a ±500 kV HVDC cable. The influence of protrusion geometry, material properties, and operating conditions was systematically investigated, leading to the following conclusions. First, protrusion height exerts the most pronounced impact on the field distortion rate compared to shape and orientation. Notably, the distortion remains invariant for a fixed inclination angle relative to the cable’s radial direction. Second, elevated activation energy intensifies electric field reversal, thereby amplifying the distortion induced by protrusions. As the activation energy rises from 0.85 eV to 1.00 eV, the distortion rate increases linearly from 9.30 to 10.38. Similarly, the distortion rate scales near-linearly as operating conditions transition from light load to full load. Overall, under steady-state DC stress, the electric field distortions exhibit significant complexity and severity, as they are inherently governed by the highly non-linear, temperature-dependent conductivity and the intense coupling of thermal and electrical fields.
