# Supplementary material for: Influence of semiconductive shielding protrusions on the electric field distribution in ±500 kV HVDC cables
Source: PLoS One. 2026 Aug 19;21(8):e0356685. doi: 10.1371/journal.pone.0356685 (PMC13489487; doi:10.1371/journal.pone.0356685)
Supplement: Supplementary file 1 [file pone.0356685.s002.docx]

**Authors’ response to comments of the reviewers**

Manuscript ID: PONE-D-26-17827

Manuscript Title: Influence of semiconductive shielding protrusions on the electric field distribution in ±500 kV HVDC cables

Dear Editor and Reviewers,

We want to thank you for your valuable comments. We submitted 4 files, including a revised manuscript, a track changes file, a response letter and an archive containing high-resolution figures. In the following, we respond to each of your concerns and recommendations. We have used different colors to highlight our responses to different reviewers, as follows:

Reviewer 1

Reviewer 2

Reviewer 3

Reviewer 1

*The paper addresses the study of the influence of protrusions on the electric field distribution within HVDC cable insulation, by considering various parameters as sizes and shapes of protrusions, activation energy of conductivity for insulation materials and temperature difference across the insulation. The results of a 3D FEM simulation model are shown. The argument is topical. The results may be of industrial interest even if only simulation is proposed. Software is not specified: please, add description of the used software (commercial or not). Some comparison with existing similar experimental results should be added, even if referred to HVDC cables not strictly corresponding to that analyzed by the authors, for validating the simulation results of the proposed cable configuration.*

**Response:** We sincerely thank the reviewer for the constructive comments and valuable suggestions regarding the software description and model validation.

Regarding the software, we have clarified in the revised manuscript in the “Model solving” section of the revised manuscript that our 3D electromagnetic-thermal coupling model was implemented and solved using a commercial software ANSYS, and the change in manuscript is located at 8 line 186.

Regarding model validation, directly and non-destructively measuring the 3D internal electric field within solid cable insulation remains a significant global technical challenge. Furthermore, experimental field measurements specifically targeting microscopic interfacial protrusions are extremely scarce in published literature. To validate our proposed cable configuration and numerical results, we compared our simulation findings with models from established research. Specifically, we verified that the localized field enhancement trends calculated by our model consistently align with the theoretical equations governing the relationship between the field distortion rate and the aspect ratio of protrusions, as thoroughly established in previous studies [[29] Gutiérrez S, Sancho I, Fontán L, De Nó J. Effect of protrusions in HVDC cables. IEEE Transactions on Dielectrics and Electrical Insulation. 2012;19(5): 1774-1781.] [[30] Bahder G, Eager GS, Silver DA, Lukac RG. Criteria for determining performance in service of crosslinked polyethylene insulated power cables. IEEE Transactions on Power Apparatus and Systems. 1976;95(5): 1552-1566.] [[31] Mukherjee M, Chatterjee S, Dalai S. Electric field enhancement in an XLPE insulated power cable containing different voids and protrusions. IET Conference Publications. 2015;2015(CP683): 220-224.] This result confirms the reliability and accuracy of our simulation framework.

We have added a dedicated new sub-section "3.1 Validation of the simulation model" at the beginning of the "Results and discussion" section to detail this validation process, which located at page 5 line 190.

Reviewer 2

1. *Lines 153–154 contain “Error! Reference source not found.”, indicating a problem with citation format or numbering. Please check and correct all references.*

**Response**: We sincerely apologize for this formatting error, which was caused by broken cross-reference links in MS Word during file conversion. We have thoroughly checked the entire manuscript and converted all reference fields to plain text to ensure this error is completely resolved.

2. *Cable geometry and material parameters are provided, but the source of these values (experimental measurement, manufacturer data, or literature) is unclear. Please clarify.*

**Response**: We appreciate the reviewer's attention to detail, and we are sorry for leaving this ambiguous. The geometry parameters and material properties are not arbitrarily assumed; The cable structure is based on production data which is used for industrial production from Chongqing Taishan Cable Co., Ltd., and the material parameters are derived from Reference [[24] Liu Y, Zhang SD, Cao XL, Zhang C, Li WP. Simulation of electric field distribution in the XLPE insulation of a 320 kV DC cable under steady and time-varying states. IEEE Transactions on Dielectrics and Electrical Insulation. 2018;25(3): 954-964.] [[25] Vu TTN, Teyssedre G, Vissouvanadin B, Le Roy S, Laurent C. Correlating conductivity and space charge measurements in multi-dielectrics under various electrical and thermal stresses. IEEE Transactions on Dielectrics and Electrical Insulation. 2015;22(1): 117-127.]. [[26] Chen G, Hao M, Xu Z, Vaughan A, Cao J, Wang H. Review of high voltage direct current cables. CSEE Journal of Power and Energy Systems. 2015;1(2): 9-21.] We have clarified the source of these parameters and added corresponding references in the revised text which located at page 3 line 82 and page 6 line 148.

3. *Qted in Eq. (5) is described as “thermoelastic damping power,” but the manuscript does not specify which part of heat generation it represents. Clarification is needed.*

**Response**: Thank you for pointing this out. $Q_{ted}$ represents the heat generated by mechanical deformation. However, since our study focuses strictly on the steady-state operation of the cable without varying mechanical loads, the heat generated by mechanical deformation is negligible, and $Q_{ted}$ is set to zero. We have added this clarification directly under Equation (5) which located at page 6 line 143.

4. *Figures (Fig. 3–Fig. 12) have low resolution; local details are difficult to see. High-resolution or vector images are recommended.图片质量】*

**Response**: We completely agree. We have re-exported and replaced Fig. 3 to Fig. 12 with high-resolution images to ensure that all local field distribution details are clearly visible.

5. *In Fig. 6(a) and Fig. 7(a), insulation layer, semiconductive layer, and interface are not labeled. Add clear layer/interface labels and explain the meaning of a/b/c axes in captions.*

*【添加标签和注释】*

**Response**: We apologize for the lack of the label which causes the misunderstanding. We have modified Fig. 6(a) and Fig. 7(a) by adding clear text labels and arrows pointing to the Main Insulation, Semiconductive Layer and the Interface. Additionally, we have updated label to define the physical meaning of the a, b and c which represent the protrusion's penetration depth, intermediate axis, and minor axis, respectively.

6. *The set temperature field (inner/outer shields, radial gradient) lacks source information. Clarify the origin or calculation basis.*

**Response**: We thank the reviewer for pointing out the initial lack of clarity regarding the temperature field boundaries. We addressed this by adding an explanatory paragraph in the Solving principles and materials section. This new text clarifies that the 70°C inner and 50°C outer boundary conditions are based on the standard full-load thermal limits of XLPE DC cables, where 70°C is the typical maximum permissible operating temperature to prevent thermal aging. To clarify the calculation basis, we have explicitly added references to the corresponding national standards [[8] China Electrical Equipment Industry Association. D.C. extruded cable systems for power transmission at a rated voltage up to and including 500kV—Part 2: D.C. land cables (GB/T 31489.2-2020). Beijing: Standards Press of China; 2020.] [[9] China Electrical Equipment Industry Association. D.C. extruded cable systems for power transmission at a rated voltage up to and including 500kV—Part 3: D.C. submarine cables (GB/T 31489.3-2020). Beijing: Standards Press of China; 2020.] [[10] China Electrical Equipment Industry Association. D.C. extruded cable systems for power transmission at a rated voltage up to and including 500kV—Part 4: Accessories for D.C. cables (GB/T 31489.4-2020). Beijing: Standards Press of China; 2020.] to support these temperature settings which located at page 7 line 164.

7. *What is the practical significance of this study? In engineering practice, a smoother interface—or no protrusions at all—is always preferred. Therefore, the investigation of protrusion height appears to have limited practical relevance. Similarly, it is already known that higher activation energy is detrimental to insulation performance.*

**Response**: We thank the reviewer for this comment. We completely agree that a perfectly smooth interface is always the ideal goal in engineering practice. However, during the actual industrial production process, due to manufacturing limitations, microscopic interfacial protrusions are inevitable. The practical significance of our study lies precisely in addressing this "non-ideal reality" by providing rigorous theoretical support for defect evaluation. Existing literature and models have shown that protrusions in the semiconductive shields of HVDC cables lead to severe localized electric field distortion. Theoretical analyses also indicate that electric field distortion is comprehensively determined by parameters such as height, aspect ratio, and spatial inclination angle. The core novelty of our study lies in establishing a comprehensive 3D electro-thermal model. We quantitatively reveal how irregular 3D geometric morphologies intricately couple with the insulation's activation energy and real-world thermal gradients to exacerbate local field distortion.

We reiterate our conclusion: it is imperative to comprehensively and holistically evaluate these multi-dimensional geometric parameters to provide meaningful guidance for practical defect control.

There are significant differences in the activation energies of various commercial materials. Furthermore, while the impact of activation energy on electric field distribution is generally not considered under AC conditions, it must be considered under DC conditions because the electrical conductivity of solid dielectrics is highly dependent on temperature under DC stress. Therefore, investigating the specific ranges within which the activation energies of different materials fall, and how they specifically exacerbate electric field distortion, is a highly important aspect of our research.

Current studies [[16] Xia J, Shi N, Sun J. Simulation of electric field distortion caused by main insulation defects morphology of XLPE power cable. Wire & Cable. 2022;6: 1-6.] [[18] Yin Y, Wu J, Hu J, Zhang L, Sun L, Shen Y. Simulation of electric field distortion caused by interface morphology between insulation and shielding in HVDC cables. Journal of Electrical Engineering. 2018;13(11): 30-36.] [[30] Bahder G, Eager GS, Silver DA, Lukac RG. Criteria for determining performance in service of crosslinked polyethylene insulated power cables. IEEE Transactions on Power Apparatus and Systems. 1976;95(5): 1552-1566.] have conducted simulation analyses on idealized spherical and spheroidal protrusions. In contrast, we considered the irregular, asymmetric 3D protrusions in real application, and effects with specific ranges of activation energy under DC thermal gradients. To maintain the conciseness of the main manuscript, we are providing this comparative discussion regarding the comparation between previous studies and our research exclusively in this response letter and have not added this additional content to the main text.

Reviewer 3

1. *The authors claim that previous studies mainly focused on 2D analyses while this work employs a 3D FEM model. However, the manuscript does not sufficiently demonstrate the practical advantages of the 3D model over existing 2D studies. It is recommended that to explain, why a 3D model is necessary, which physical phenomena cannot be captured by 2D models, quantitative differences between 2D and 3D results and additional engineering insights obtained from the 3D analysis.*

**Response**: We sincerely appreciate the reviewer's insightful comments regarding the necessity and practical advantages of the 3D model. 2D axisymmetric models are mathematically restricted to symmetric defects. However, during actual manufacturing processes, semiconductive protrusions exhibit asymmetrical cross-sections and varying inclination angles. These characteristics cannot be established in 2D simulations, where dimensional variations are strictly limited to height and width. In contrast, our 3D model can capture dimensional differences across two cross-sectional directions.

Quantitatively, our 3D analysis demonstrates that altering the cross-sectional asymmetry or varying the inclination angle from 0° to 90° can reduce the electric field distortion rate by over 50%, specifically dropping from 9.78 to 4.11. This significant variation is a physical phenomenon completely unobservable in idealized 2D models. To elucidate the points, we have added a clarifying paragraph in the newly created sub-section "3.1 Validation of the simulation model" at the beginning of the "Results and discussion" section which located at page 8 line 190.

2. *The entire study is based on numerical simulations, no experimental measurements, laboratory testing, or comparison with published experimental data are provided to validate the model. It is recommended that to compare the simulated electric field distributions with available literature results. Provide validation against experimental breakdown data or field measurements if available. Discuss model accuracy and limitations.*

**Response**: We sincerely thank the reviewer for emphasizing the importance of model validation and for suggesting a detailed discussion on the model's accuracy and limitations.

As non-destructively measuring the 3D internal electric field within solid XLPE insulation remains a significant technical challenge, direct experimental field measurements targeting microscopic interfacial protrusions are extremely scarce. Therefore, to ensure the validity of our model, we compared our simulation results with the model developed by Gutiérrez S [[29] Gutiérrez S, Sancho I, Fontán L, De Nó J. Effect of protrusions in HVDC cables. IEEE Transactions on Dielectrics and Electrical Insulation. 2012;19(5): 1774-1781.]. This consistency with classical physical laws confirms the baseline accuracy and reliability of our numerical settings.

While the current model effectively evaluates the steady-state DC field distortion governed by highly non-linear, temperature-dependent conductivity, it simplifies the time-dependent accumulation process of space charge.

We have integrated these discussions into the newly added sub-section "3.1 Validation of the simulation model" at the beginning of the "Results and discussion" section which located at page 8 line 190.

3. *The FEM model description is incomplete, and important information is missing, including, software platform used, number of mesh elements, mesh independence study, convergence criteria, solver settings, and computational accuracy.*

**Response**: We sincerely appreciate the reviewer for pointing out the incomplete description of the finite element model. The 3D model was established using the commercial software ANSYS which has now been specified in the revised manuscript page 8 line 186. The steady-state calculation used a fully coupled linear solver with an automatic nonlinear method. To guarantee solver stability, the initial damping factor was set to 1, the minimum damping factor to 1e-4, and an automatic recovered damping factor was enabled with a recovery factor of 0.75. The update step limit and step growth limit were set to 10 and 1, respectively. The convergence criteria were based on tolerance and residual termination techniques, with a maximum of 25 iterations, a tolerance factor of 1, and a residual factor of 1000. Regarding the mesh generation, the computational domain consists of 1,481,522 tetrahedral elements, 91,398 triangular elements, 2,321 edge elements, and 41 vertex elements. The average element quality reached 0.6953 with a minimum quality of 0.2791, exhibiting excellent geometric characteristics. Meanwhile, a rigorous mesh independence study was conducted: mesh independence was confirmed when the relative change of the target physical quantity between adjacent mesh densities was less than 1%, and this variation was strictly controlled to within 0.1% for regions with high accuracy requirements. Ultimately, the mesh quality was excellent, the residual curve exhibited smooth convergence, and the fluctuation of the monitored quantities at fixed points remained below 0.5%, indicating good numerical stability and convergence.

4. *The protrusion height varies from 50 μm to 300 μm, manuscript does not explain, whether these dimensions are representative of manufacturing defects observed in industrial HVDC cables. The authors should provide references or industrial evidence supporting the selected defect sizes.*

**Response:** We thank the reviewer for highlighting the importance of justifying our simulation parameters with industrial evidence. To address this, we added citations to authoritative industrial standards [[8] China Electrical Equipment Industry Association. D.C. extruded cable systems for power transmission at a rated voltage up to and including 500kV—Part 2: D.C. land cables (GB/T 31489.2-2020). Beijing: Standards Press of China; 2020.] [[9] China Electrical Equipment Industry Association. D.C. extruded cable systems for power transmission at a rated voltage up to and including 500kV—Part 3: D.C. submarine cables (GB/T 31489.3-2020). Beijing: Standards Press of China; 2020.] [[10] China Electrical Equipment Industry Association. D.C. extruded cable systems for power transmission at a rated voltage up to and including 500kV—Part 4: Accessories for D.C. cables (GB/T 31489.4-2020). Beijing: Standards Press of China; 2020.] [[11] CIGRE Working Group B1.32. Recommendations for testing DC extruded cable systems for power transmission at a rated voltage up to 500 kV (Brochure CIGRE 496). CIGRE; 2012.]. The revised text clarifies that while high-quality manufacturing guidelines aim to strictly limit protrusions below 50 μm, agglomeration of semiconductive particles or extrusion instability during suboptimal processing can occasionally induce severe defects reaching 200-300 μm. Therefore, our simulated range effectively covers both acceptable manufacturing tolerances and critical industrial defect scenarios. This change is located at page 4 line 90.

5. *The authors should clarify what distortion-rate thresholds are considered dangerous. Whether a distortion rate of 9.78 or 102.04 corresponds to insulation failure risk. How these values relate to breakdown strength of XLPE.*

**Response**: We are very grateful for this valuable comment, which helps bridge our numerical findings with practical engineering safety. In the revised manuscript, based on the intrinsic DC breakdown strength of commercial XLPE and the definition in Equation (7), we have explicitly defined a dangerous distortion-rate threshold, referred to as Equation (8). According to the study by Mazzanti G et al. [[32] Mazzanti G, Montanari GC, Dissado LA. Electrical aging and life models: the role of space charge. IEEE Transactions on Dielectrics and Electrical Insulation. 2005;12(5): 876-890.], the intrinsic DC breakdown strength of solid polymeric insulation typically ranges from 100 to 500 kV/mm. Based on the definition of distortion rate in our study, the critical condition for insulation failure occurs when the maximum localized electric field $E_{bd,min}$ reaches the lower limit of the material's breakdown strength. Under full-load conditions, the average electric field $E_{av}$ across the insulation is approximately 16.6 kV/mm. Therefore, the critical distortion-rate threshold $\alpha_{critical}$ can be calculated as: 说明

$$\alpha_{critical}=\frac{E_{max(critical)}}{E_{av}}=\frac{E_{bd,min}}{E_{av}}=\frac{100}{16.6}\approx6.0$$

The simulated distortion rate of $\alpha=9.78$ exceeds $\alpha_{critical}$. It amplifies the local electric field to 163 kV/mm, which safely exceeds the 100 kV/mm minimum breakdown threshold, and is thus explicitly classified as dangerous, indicating a tangible risk of insulation failure. An extreme distortion rate of $\alpha=102.04$ implies a localized electric field exceeding 300 kV/mm, carrying a risk of immediate breakdown. Furthermore, we explained in the manuscript that beyond the immediate failure risks, long-term exposure to these super-threshold fields significantly accelerates electrical treeing and aging according to the classical inverse power law, thereby prematurely terminating the service life of the HVDC cable. This revision is located at page 10 line 252.

6. *Activation Energy Analysis Requires Deeper Discussion*

*The manuscript reports that the distortion rate increases with activation energy.*

*It is recommended that to explain, the conductivity mechanism responsible for this trend. Whether the activation energy range (0.85–1.00 eV) reflects commercial XLPE materials and practical implications for insulation material selection.*

**Response**: We sincerely thank the reviewer for emphasizing the need to elaborate on the underlying physical mechanism behind the activation energy trends.

Under high DC stress and thermal gradients, steady-state conduction in commercial XLPE is predominantly governed by ionic conduction and carrier hopping. The activation energy represents the potential barrier that these ions or charge carriers must overcome to migrate through the polymer matrix. Consequently, a higher activation energy makes the carrier mobility and the macroscopic conductivity highly dependent on temperature. Because the outer shield interface operates at a lower temperature than the inner conductor, the local conductivity drops much more sharply for materials with high activation energy. Under steady-state DC stress, the electric field distribution is inversely proportional to conductivity, thereby exacerbating the electric field concentration at the colder outer protrusion.

For the simulated activation energy range of 0.85-1.00 eV, we adopted the range investigated in the study by Liu Y et al. The materials and parameters selected by Liu Y were based on an actual commercial 320 kV DC XLPE cable manufactured by Zhongtian Technology Company. [[24] Liu Y, Zhang SD, Cao XL, Zhang C, Li WP. Simulation of electric field distribution in the XLPE insulation of a 320 kV DC cable under steady and time-varying states. IEEE Transactions on Dielectrics and Electrical Insulation. 2018;25(3): 954-964] [[25] Vu TTN, Teyssedre G, Vissouvanadin B, Le Roy S, Laurent C. Correlating conductivity and space charge measurements in multi-dielectrics under various electrical and thermal stresses. IEEE Transactions on Dielectrics and Electrical Insulation. 2015;22(1): 117-127.] [[26] Chen G, Hao M, Xu Z, Vaughan A, Cao J, Wang H. Review of high voltage direct current cables. CSEE Journal of Power and Energy Systems. 2015;1(2): 9-21. By utilizing the specific parameters of a commercial product, we ensured that this study accurately reflects practical industrial properties. From an engineering perspective, this finding explicitly guides manufacturers to favor lower activation energies when selecting or developing insulation materials to mitigate local electric field distortion and improve the overall reliability of the cable. This change is located at page 6 line 149.

7. *In the Conclusion "Field distortions under DC stress are more complex and severe than those under AC conditions" is not directly demonstrated in the manuscript because no AC simulations are presented. Either provide AC simulation results for comparison, or rephrase this statement to avoid unsupported conclusions.*

**Response**: We sincerely appreciate the reviewer's rigorous critique. Initially, we had planned to include comparative simulations under AC conditions. However, considering the length constraints of the manuscript, we ultimately removed the content related to AC simulations. We have revised the relevant statements in the "Conclusion" section. We removed the unsupported comparisons with AC conditions and focused purely on describing the DC electric field distortion which located at page 15 line 382.

8. *Typographical and Language Issues*

*Several grammatical and wording issues should be corrected, examples include:*

*i. "insulation system" → "an insulation system"*

*ii. "the four innermost layers of the cable is considered" → "are considered"*

*iii. "b increase" → "b increases"*

*iv. "field strength frops" → "field strength drops"*

*A thorough English language revision is recommended.*

**Response**: We are very grateful to the reviewer for the meticulous reading and for pointing out these specific language issues. We have corrected all the noted typographical and grammatical errors. Furthermore, the entire manuscript has undergone a comprehensive English language revision to ensure clarity, grammatical correctness, and academic fluency throughout.

9. *Several references appear incomplete or inconsistent. Reference [6] lacks complete bibliographic information, reference [15] appears incomplete, some conference references do not contain page numbers. The reference list should be carefully checked according to journal guidelines.*

**Response**: We thank the reviewer for the rigorous check on our reference list. To ensure compliance with the journal guidelines, we have thoroughly reviewed the bibliography and updated the incomplete entries with full bibliographic details. We also verified that all cited conference proceedings now include their appropriate page numbers.

10. *Some symbols are inconsistently defined.*

*i. Conductivity is denoted using both σ and γ.*

*ii. Relative permittivity values in Table 2 appear unusual and should be clarified.*

*iii. Units and notation should be standardized throughout the manuscript.*

**Response**: We appreciate the reviewer’s careful check regarding our notation and data representation. To resolve these inconsistencies, we have implemented the following corrections:

First, we have standardized the conductivity notation to $\sigma$ throughout the entire manuscript, replacing conflicting instances of $\gamma$.

Second, we have updated Table 2 and added corresponding clarifications in the text. Since copper is a metallic conductor, its static relative permittivity has been corrected to N/A. Furthermore, the high relative permittivity value of 1000 for the semiconductive shielding layer has been clarified; this is a well-documented phenomenon caused by the heavy carbon black loading near the percolation threshold [[27] Dang ZM, Yuan JK, Zha JW, Zhou T, Li ST, Hu GH. Fundamentals, processes and applications of high-permittivity polymer-matrix composites. Progress in Materials Science. 2012;57(4): 660-723.].

Third, we added a clarification in the manuscript which located at page 7 line 157: our study evaluates steady-state DC conditions, the electric field distribution is mathematically governed exclusively by the non-linear conductivity matrix. Relative permittivity does not participate in the steady-state potential solving equations; hence, this parameter does not alter any of the simulated outcomes.

Finally, all units and notations have been thoroughly reviewed and standardized across the manuscript.
