# Supplementary material for: Influence of semiconductive shielding protrusions on the electric field distribution in ±500 kV HVDC cables
Source: PLoS One. 2026 Aug 19;21(8):e0356685. doi: 10.1371/journal.pone.0356685 (PMC13489487; doi:10.1371/journal.pone.0356685)
Supplement: Peer review history

# Peer review: Decision Letter 0

**DOI:** 10.1371/journal.pone.0356685.r001

**Authors:** Azim Uddin

27 May 2026

Dear Dr. Xu,

Thank you for submitting your manuscript to PLOS ONE. After careful consideration, we feel that it has merit but does not fully meet PLOS ONE’s publication criteria as it currently stands. Therefore, we invite you to submit a revised version of the manuscript that addresses the points raised during the review process.

Please submit your revised manuscript by Jul 11 2026 11:59PM. If you will need more time than this to complete your revisions, please reply to this message or contact the journal office at plosone@plos.org. When you're ready to submit your revision, log on to https://www.editorialmanager.com/pone/ and select the 'Submissions Needing Revision' folder to locate your manuscript file.

- A letter that responds to each point raised by the academic editor and reviewer(s). You should upload this letter as a separate file labeled 'Response to Reviewers'.
- A marked-up copy of your manuscript that highlights changes made to the original version. You should upload this as a separate file labeled 'Revised Manuscript with Track Changes'.
- An unmarked version of your revised paper without tracked changes. You should upload this as a separate file labeled 'Manuscript'.

If you would like to make changes to your financial disclosure, please include your updated statement in your cover letter. Guidelines for resubmitting your figure files are available below the reviewer comments at the end of this letter.

If applicable, we recommend that you deposit your laboratory protocols in protocols.io to enhance the reproducibility of your results. Protocols.io assigns your protocol its own identifier (DOI) so that it can be cited independently in the future. For instructions see: https://journals.plos.org/plosone/s/submission-guidelines#loc-laboratory-protocols. Additionally, PLOS ONE offers an option for publishing peer-reviewed Lab Protocol articles, which describe protocols hosted on protocols.io. Read more information on sharing protocols at https://plos.org/protocols?utm_medium=editorial-email&utm_source=authorletters&utm_campaign=protocols.

As the corresponding author, your ORCID iD is verified in the submission system and will appear in the published article. PLOS supports the use of ORCID, and we encourage all coauthors to register for an ORCID iD and use it as well. Please encourage your coauthors to verify their ORCID iD within the submission system before final acceptance, as unverified ORCID iDs will not appear in the published article. *Only*  the individual author can complete the verification step; PLOS staff *cannot*  verify ORCID iDs on behalf of authors.

We look forward to receiving your revised manuscript.

Kind regards,

Azim Uddin, Ph.D.

Academic Editor

PLOS One

Journal Requirements:

When submitting your revision, we need you to address these additional requirements.

1. Please ensure that your manuscript meets PLOS ONE's style requirements, including those for file naming. The PLOS ONE style templates can be found at https://journals.plos.org/plosone/s/file?id=wjVg/PLOSOne_formatting_sample_main_body.pdf and https://journals.plos.org/plosone/s/file?id=ba62/PLOSOne_formatting_sample_title_authors_affiliations.pdf

2. During your revisions, please note that a simple title correction is required: Kindly ensure to remove "'Date: March 28, 2026 To: Editor, PLOS ONE'" from the title of online submission form. Please ensure this is updated in the manuscript file and the online submission information.

3. Please note that PLOS One has specific guidelines on code sharing for submissions in which author-generated code underpins the findings in the manuscript. In these cases, we expect all author-generated code to be made available without restrictions upon publication of the work. Please review our guidelines at https://journals.plos.org/plosone/s/materials-and-software-sharing#loc-sharing-code and ensure that your code is shared in a way that follows best practice and facilitates reproducibility and reuse.

4. We suggest you thoroughly copyedit your manuscript for language usage, spelling, and grammar. If you do not know anyone who can help you do this, you may wish to consider employing a professional scientific editing service.

The American Journal Experts (AJE) (https://www.aje.com/) is one such service that has extensive experience helping authors meet PLOS guidelines and can provide language editing, translation, manuscript formatting, and figure formatting to ensure your manuscript meets our submission guidelines. Please note that having the manuscript copyedited by AJE or any other editing services does not guarantee selection for peer review or acceptance for publication.

Upon resubmission, please provide the following:

- The name of the colleague or the details of the professional service that edited your manuscript

- A copy of your manuscript showing your changes by either highlighting them or using track changes (uploaded as a *supporting information* file)

- A clean copy of the edited manuscript (uploaded as the new *manuscript* file)

5. Thank you for stating in your Funding Statement:

[This work was supported by Fundamental Research Funds for the Central Universities (grant number SWU120069) and the Natural Science Foundation of Chongqing, China (grant number sl202100000470).].

Please provide an amended statement that declares *all* the funding or sources of support (whether external or internal to your organization) received during this study, as detailed online in our guide for authors at http://journals.plos.org/plosone/s/submit-now.  Please also include the statement “There was no additional external funding received for this study.” in your updated Funding Statement.

Please include your amended Funding Statement within your cover letter. We will change the online submission form on your behalf.

6. Thank you for stating the following in the Competing Interests section:

[NO authors have competing interests].

We note that one or more of the authors are employed by a commercial company: China Southern Power Grid Co Ltd.

1. Please provide an amended Funding Statement declaring this commercial affiliation, as well as a statement regarding the Role of Funders in your study. If the funding organization did not play a role in the study design, data collection and analysis, decision to publish, or preparation of the manuscript and only provided financial support in the form of authors' salaries and/or research materials, please review your statements relating to the author contributions, and ensure you have specifically and accurately indicated the role(s) that these authors had in your study. You can update author roles in the Author Contributions section of the online submission form.

Please also include the following statement within your amended Funding Statement.

“The funder provided support in the form of salaries for authors [insert relevant initials], but did not have any additional role in the study design, data collection and analysis, decision to publish, or preparation of the manuscript. The specific roles of these authors are articulated in the ‘author contributions’ section.”

If your commercial affiliation did play a role in your study, please state and explain this role within your updated Funding Statement.

2. Please also provide an updated Competing Interests Statement declaring this commercial affiliation along with any other relevant declarations relating to employment, consultancy, patents, products in development, or marketed products, etc.

Within your Competing Interests Statement, please confirm that this commercial affiliation does not alter your adherence to all PLOS ONE policies on sharing data and materials by including the following statement: "This does not alter our adherence to PLOS ONE policies on sharing data and materials.” (as detailed online in our guide for authors http://journals.plos.org/plosone/s/competing-interests) . If this adherence statement is not accurate and there are restrictions on sharing of data and/or materials, please state these. Please note that we cannot proceed with consideration of your article until this information has been declared.

Please include both an updated Funding Statement and Competing Interests Statement in your cover letter. We will change the online submission form on your behalf.

7. If your research concerns only data provided within your submission, please write "All data are in the manuscript and/or supporting information files" as your Data Availability Statement.

If the reviewer comments include a recommendation to cite specific previously published works, please review and evaluate these publications to determine whether they are relevant and should be cited. There is no requirement to cite these works unless the editor has indicated otherwise.

[Note: HTML markup is below. Please do not edit.]

Reviewers' comments:

Reviewer's Responses to Questions

**Comments to the Author**

1. Is the manuscript technically sound, and do the data support the conclusions?

Reviewer #1: Partly

Reviewer #2: Yes

Reviewer #3: Partly

**********

2. Has the statistical analysis been performed appropriately and rigorously?

Reviewer #1: N/A

Reviewer #2: Yes

Reviewer #3: Yes

**********

3. Have the authors made all data underlying the findings in their manuscript fully available?

The PLOS Data policy

Reviewer #1: No

Reviewer #2: Yes

Reviewer #3: No

**********

4. Is the manuscript presented in an intelligible fashion and written in standard English?

Reviewer #1: Yes

Reviewer #2: Yes

Reviewer #3: Yes

**********

Reviewer #1: The paper addresses the study of the influence of protrusions on the electric field distribution within HVDC cable insulation, by considering various parameters as sizes and shapes of protrusions, activation energy of conductivity for insulation materials and temperature difference across the insulation. The results of a 3D FEM simulation model are shown. The argument is topical. The results may be of industrial interest even if only simulation is proposed. Software is not specified: please, add description of the used software (commercial or not). Some comparison with existing similar experimental results should be added, even if referred to HVDC cables not strictly corresponding to that analysed by the authors, for validating the simulation results of the proposed cable configuration.

Reviewer #2: The detailed comments are as follows:

1. Lines 153–154 contain “Error! Reference source not found.”, indicating a problem with citation format or numbering. Please check and correct all references.

2. Cable geometry and material parameters are provided, but the source of these values (experimental measurement, manufacturer data, or literature) is unclear. Please clarify.

3. Qted in Eq. (5) is described as “thermoelastic damping power,” but the manuscript does not specify which part of heat generation it represents. Clarification is needed.

4. Figures (Fig. 3–Fig. 12) have low resolution; local details are difficult to see. High-resolution or vector images are recommended.

5. In Fig. 6(a) and Fig. 7(a), insulation layer, semiconductive layer, and interface are not labeled. Add clear layer/interface labels and explain the meaning of a/b/c axes in captions.

6. The set temperature field (inner/outer shields, radial gradient) lacks source information. Clarify the origin or calculation basis.

7. What is the practical significance of this study? In engineering practice, a smoother interface—or no protrusions at all—is always preferred. Therefore, the investigation of protrusion height appears to have limited practical relevance. Similarly, it is already known that higher activation energy is detrimental to insulation performance.

Reviewer #3: The manuscript investigates the influence of semiconductive shielding protrusions on electric field distribution in ±500 kV HVDC cables using a 3D finite element model. The topic is relevant to HVDC cable reliability, and the manuscript provides useful parametric analyses regarding protrusion geometry, activation energy, and thermal gradients. However, the work remains largely simulation-based and requires stronger validation, clearer methodological descriptions, and deeper discussion of engineering implications. In its current form, the scientific contribution is somewhat incremental relative to previous studies on HVDC cable interface defects.

1. The authors claim that previous studies mainly focused on 2D analyses while this work employs a 3D FEM model. However, the manuscript does not sufficiently demonstrate the practical advantages of the 3D model over existing 2D studies. It is recommended that to explain, why a 3D model is necessary, which physical phenomena cannot be captured by 2D models, quantitative differences between 2D and 3D results and additional engineering insights obtained from the 3D analysis.

2. The entire study is based on numerical simulations, no experimental measurements, laboratory testing, or comparison with published experimental data are provided to validate the model. It is recommended that to compare the simulated electric field distributions with available literature results. Provide validation against experimental breakdown data or field measurements if available. Discuss model accuracy and limitations.

3. The FEM model description is incomplete, and important information is missing, including, software platform used, number of mesh elements, mesh independence study, convergence criteria, solver settings, and computational accuracy.

4. The protrusion height varies from 50 μm to 300 μm, manuscript does not explain, whether these dimensions are representative of manufacturing defects observed in industrial HVDC cables. The authors should provide references or industrial evidence supporting the selected defect sizes.

5. The authors should clarify what distortion-rate thresholds are considered dangerous. Whether a distortion rate of 9.78 or 102.04 corresponds to insulation failure risk. How these values relate to breakdown strength of XLPE.

6. Activation Energy Analysis Requires Deeper Discussion

The manuscript reports that the distortion rate increases with activation energy.

It is recommended that to explain, the conductivity mechanism responsible for this trend. Whether the activation energy range (0.85–1.00 eV) reflects commercial XLPE materials and practical implications for insulation material selection.

7. In the Conclusion "Field distortions under DC stress are more complex and severe than those under AC conditions" is not directly demonstrated in the manuscript because no AC simulations are presented. Either provide AC simulation results for comparison, or rephrase this statement to avoid unsupported conclusions.

8. Typographical and Language Issues

Several grammatical and wording issues should be corrected, examples include:

i. "insulation system" → "an insulation system"

ii. "the four innermost layers of the cable is considered" → "are considered"

iii. "b increase" → "b increases"

iv. "field strength frops" → "field strength drops"

A thorough English language revision is recommended.

9. Several references appear incomplete or inconsistent. Reference [6] lacks complete bibliographic information, reference [15] appears incomplete, some conference references do not contain page numbers. The reference list should be carefully checked according to journal guidelines.

10. Some symbols are inconsistently defined.

i. Conductivity is denoted using both σ and γ.

ii. Relative permittivity values in Table 2 appear unusual and should be clarified.

iii. Units and notation should be standardized throughout the manuscript.

**********

what does this mean?). If published, this will include your full peer review and any attached files.

If you choose “no”, your identity will remain anonymous but your review may still be made public.

**Do you want your identity to be public for this peer review?**  For information about this choice, including consent withdrawal, please see our Privacy Policy

Reviewer #1: No

Reviewer #2: No

Reviewer #3: **Yes:**  Dr. Muhammad Wahab Hanif

**********

[NOTE: If reviewer comments were submitted as an attachment file, they will be attached to this email and accessible via the submission site. Please log into your account, locate the manuscript record, and check for the action link "View Attachments". If this link does not appear, there are no attachment files.]

To ensure your figures meet our technical requirements, please review our figure guidelines: https://journals.plos.org/plosone/s/figures

You may also use PLOS’s free figure tool, NAAS, to help you prepare publication quality figures: https://journals.plos.org/plosone/s/figures#loc-tools-for-figure-preparation.

NAAS will assess whether your figures meet our technical requirements by comparing each figure against our figure specifications.

# Author comment: Author response to Decision Letter 1

**DOI:** 10.1371/journal.pone.0356685.r002

17 Jul 2026

Authors’ response to comments of the reviewers

Manuscript ID: PONE-D-26-17827

Manuscript Title: Influence of semiconductive shielding protrusions on the electric field distribution in ±500 kV HVDC cables

Dear Editor and Reviewers,

We want to thank you for your valuable comments. We submitted 4 files, including a revised manuscript, a track changes file, a response letter and an archive containing high-resolution figures. In the following, we respond to each of your concerns and recommendations. We have used different colors to highlight our responses to different reviewers, as follows:

Reviewer 1

Reviewer 2

Reviewer 3

Reviewer 1

The paper addresses the study of the influence of protrusions on the electric field distribution within HVDC cable insulation, by considering various parameters as sizes and shapes of protrusions, activation energy of conductivity for insulation materials and temperature difference across the insulation. The results of a 3D FEM simulation model are shown. The argument is topical. The results may be of industrial interest even if only simulation is proposed. Software is not specified: please, add description of the used software (commercial or not). Some comparison with existing similar experimental results should be added, even if referred to HVDC cables not strictly corresponding to that analyzed by the authors, for validating the simulation results of the proposed cable configuration.

Response: We sincerely thank the reviewer for the constructive comments and valuable suggestions regarding the software description and model validation.

Regarding the software, we have clarified in the revised manuscript in the “Model solving” section of the revised manuscript that our 3D electromagnetic-thermal coupling model was implemented and solved using a commercial software ANSYS, and the change in manuscript is located at 8 line 186.

Regarding model validation, directly and non-destructively measuring the 3D internal electric field within solid cable insulation remains a significant global technical challenge. Furthermore, experimental field measurements specifically targeting microscopic interfacial protrusions are extremely scarce in published literature. To validate our proposed cable configuration and numerical results, we compared our simulation findings with models from established research. Specifically, we verified that the localized field enhancement trends calculated by our model consistently align with the theoretical equations governing the relationship between the field distortion rate and the aspect ratio of protrusions, as thoroughly established in previous studies [[29] Gutiérrez S, Sancho I, Fontán L, De Nó J. Effect of protrusions in HVDC cables. IEEE Transactions on Dielectrics and Electrical Insulation. 2012;19(5): 1774-1781.] [[30] Bahder G, Eager GS, Silver DA, Lukac RG. Criteria for determining performance in service of crosslinked polyethylene insulated power cables. IEEE Transactions on Power Apparatus and Systems. 1976;95(5): 1552-1566.] [[31] Mukherjee M, Chatterjee S, Dalai S. Electric field enhancement in an XLPE insulated power cable containing different voids and protrusions. IET Conference Publications. 2015;2015(CP683): 220-224.] This result confirms the reliability and accuracy of our simulation framework.

We have added a dedicated new sub-section "3.1 Validation of the simulation model" at the beginning of the "Results and discussion" section to detail this validation process, which located at page 5 line 190.

Reviewer 2

1. Lines 153–154 contain “Error! Reference source not found.”, indicating a problem with citation format or numbering. Please check and correct all references.

Response: We sincerely apologize for this formatting error, which was caused by broken cross-reference links in MS Word during file conversion. We have thoroughly checked the entire manuscript and converted all reference fields to plain text to ensure this error is completely resolved.

2. Cable geometry and material parameters are provided, but the source of these values (experimental measurement, manufacturer data, or literature) is unclear. Please clarify.

Response: We appreciate the reviewer's attention to detail, and we are sorry for leaving this ambiguous. The geometry parameters and material properties are not arbitrarily assumed; The cable structure is based on production data which is used for industrial production from Chongqing Taishan Cable Co., Ltd., and the material parameters are derived from Reference [[24] Liu Y, Zhang SD, Cao XL, Zhang C, Li WP. Simulation of electric field distribution in the XLPE insulation of a 320 kV DC cable under steady and time-varying states. IEEE Transactions on Dielectrics and Electrical Insulation. 2018;25(3): 954-964.] [[25] Vu TTN, Teyssedre G, Vissouvanadin B, Le Roy S, Laurent C. Correlating conductivity and space charge measurements in multi-dielectrics under various electrical and thermal stresses. IEEE Transactions on Dielectrics and Electrical Insulation. 2015;22(1): 117-127.]. [[26] Chen G, Hao M, Xu Z, Vaughan A, Cao J, Wang H. Review of high voltage direct current cables. CSEE Journal of Power and Energy Systems. 2015;1(2): 9-21.] We have clarified the source of these parameters and added corresponding references in the revised text which located at page 3 line 82 and page 6 line 148.

3. Qted in Eq. (5) is described as “thermoelastic damping power,” but the manuscript does not specify which part of heat generation it represents. Clarification is needed.

Response: Thank you for pointing this out. Q_ted represents the heat generated by mechanical deformation. However, since our study focuses strictly on the steady-state operation of the cable without varying mechanical loads, the heat generated by mechanical deformation is negligible, and Q_ted is set to zero. We have added this clarification directly under Equation (5) which located at page 6 line 143.

4. Figures (Fig. 3–Fig. 12) have low resolution; local details are difficult to see. High-resolution or vector images are recommended.图片质量】

Response: We completely agree. We have re-exported and replaced Fig. 3 to Fig. 12 with high-resolution images to ensure that all local field distribution details are clearly visible.

5. In Fig. 6(a) and Fig. 7(a), insulation layer, semiconductive layer, and interface are not labeled. Add clear layer/interface labels and explain the meaning of a/b/c axes in captions.

【添加标签和注释】

Response: We apologize for the lack of the label which causes the misunderstanding. We have modified Fig. 6(a) and Fig. 7(a) by adding clear text labels and arrows pointing to the Main Insulation, Semiconductive Layer and the Interface. Additionally, we have updated label to define the physical meaning of the a, b and c which represent the protrusion's penetration depth, intermediate axis, and minor axis, respectively.

6. The set temperature field (inner/outer shields, radial gradient) lacks source information. Clarify the origin or calculation basis.

Response: We thank the reviewer for pointing out the initial lack of clarity regarding the temperature field boundaries. We addressed this by adding an explanatory paragraph in the Solving principles and materials section. This new text clarifies that the 70°C inner and 50°C outer boundary conditions are based on the standard full-load thermal limits of XLPE DC cables, where 70°C is the typical maximum permissible operating temperature to prevent thermal aging. To clarify the calculation basis, we have explicitly added references to the corresponding national standards [[8] China Electrical Equipment Industry Association. D.C. extruded cable systems for power transmission at a rated voltage up to and including 500kV—Part 2: D.C. land cables (GB/T 31489.2-2020). Beijing: Standards Press of China; 2020.] [[9] China Electrical Equipment Industry Association. D.C. extruded cable systems for power transmission at a rated voltage up to and including 500kV—Part 3: D.C. submarine cables (GB/T 31489.3-2020). Beijing: Standards Press of China; 2020.] [[10] China Electrical Equipment Industry Association. D.C. extruded cable systems for power transmission at a rated voltage up to and including 500kV—Part 4: Accessories for D.C. cables (GB/T 31489.4-2020). Beijing: Standards Press of China; 2020.] to support these temperature settings which located at page 7 line 164.

7. What is the practical significance of this study? In engineering practice, a smoother interface—or no protrusions at all—is always preferred. Therefore, the investigation of protrusion height appears to have limited practical relevance. Similarly, it is already known that higher activation energy is detrimental to insulation performance.

Response: We thank the reviewer for this comment. We completely agree that a perfectly smooth interface is always the ideal goal in engineering practice. However, during the actual industrial production process, due to manufacturing limitations, microscopic interfacial protrusions are inevitable. The practical significance of our study lies precisely in addressing this "non-ideal reality" by providing rigorous theoretical support for defect evaluation. Existing literature and models have shown that protrusions in the semiconductive shields of HVDC cables lead to severe localized electric field distortion. Theoretical analyses also indicate that electric field distortion is comprehensively determined by parameters such as height, aspect ratio, and spatial inclination angle. The core novelty of our study lies in establishing a comprehensive 3D electro-thermal model. We quantitatively reveal how irregular 3D geometric morphologies intricately couple with the insulation's activation energy and real-world thermal gradients to exacerbate local field distortion.

We reiterate our conclusion: it is imperative to comprehensively and holistically evaluate these multi-dimensional geometric parameters to provide meaningful guidance for practical defect control.

There are significant differences in the activation energies of various commercial materials. Furthermore, while the impact of activation energy on electric field distribution is generally not considered under AC conditions, it must be considered under DC conditions because the electrical conductivity of solid dielectrics is highly dependent on temperature under DC stress. Therefore, investigating the specific ranges within which the activation energies of different materials fall, and how they specifically exacerbate electric field distortion, is a highly important aspect of our research.

Current studies [[16] Xia J, Shi N, Sun J. Simulation of electric field distortion caused by main insulation defects morphology of XLPE power cable. Wire & Cable. 2022;6: 1-6.] [[18] Yin Y, Wu J, Hu J, Zhang L, Sun L, Shen Y. Simulation of electric field distortion caused by interface morphology between insulation and shielding in HVDC cables. Journal of Electrical Engineering. 2018;13(11): 30-36.] [[30] Bahder G, Eager GS, Silver DA, Lukac RG. Criteria for determining performance in service of crosslinked polyethylene insulated power cables. IEEE Transactions on Power Apparatus and Systems. 1976;95(5): 1552-1566.] have conducted simulation analyses on idealized spherical and spheroidal protrusions. In contrast, we considered the irregular, asymmetric 3D protrusions in real application, and effects with specific ranges of activation energy under DC thermal gradients. To maintain the conciseness of the main manuscript, we are providing this comparative discussion regarding the comparation between previous studies and our research exclusively in this response letter and have not added this additional content to the main text.

Reviewer 3

1. The authors claim that previous studies mainly focused on 2D analyses while this work employs a 3D FEM model. However, the manuscript does not sufficiently demonstrate the practical advantages of the 3D model over existing 2D studies. It is recommended that to explain, why a 3D model is necessary, which physical phenomena cannot be captured by 2D models, quantitative differences between 2D and 3D results and additional engineering insights obtained from the 3D analysis.

Response: We sincerely appreciate the reviewer's insightful comments regarding the necessity and practical advantages of the 3D model. 2D axisymmetric models are mathematically restricted to symmetric defects. However, during actual manufacturing processes, semiconductive protrusions exhibit asymmetrical cross-sections and varying inclination angles. These characteristics cannot be established in 2D simulations, where dimensional variations are strictly limited to height and width. In contrast, our 3D model can capture dimensional differences across two cross-sectional directions.

Quantitatively, our 3D analysis demonstrates that altering the cross-sectional asymmetry or varying the inclination angle from 0° to 90° can reduce the electric field distortion rate by over 50%, specifically dropping from 9.78 to 4.11. This significant variation is a physical phenomenon completely unobservable in idealized 2D models. To elucidate the points, we have added a clarifying paragraph in the newly created sub-section "3.1 Validation of the simulation model" at the beginning of the "Results and discussion" section which located at page 8 line 190.

2. The entire study is based on numerical simulations, no experimental measurements, laboratory testing, or comparison with published experimental data are provided to validate the model. It is recommended that to compare the simulated electric field distributions with available literature results. Provide validation against experimental breakdown data or field measurements if available. Discuss model accuracy and limitations.

Response: We sincerely thank the reviewer for emphasizing the importance of model validation and for suggesting a detailed discussion on the model's accuracy and limitations.

As non-destructively measuring the 3D internal electric field within solid XLPE insulation remains a significant technical challenge, direct experimental field measurements targeting microscopic interfacial protrusions are extremely scarce. Therefore, to ensure the validity of our model, we compared our simulation results with the model developed by Gutiérrez S [[29] Gutiérrez S, Sancho I, Fontán L, De Nó J. Effect of protrusions in HVDC cables. IEEE Transactions on Dielectrics and Electrical Insulation. 2012;19(5): 1774-1781.]. This consistency with classical physical laws confirms the baseline accuracy and reliability of our numerical settings.

While the current model effectively evaluates the steady-state DC field distortion governed by highly non-linear, temperature-dependent conductivity, it simplifies the time-dependent accumulation process of space charge.

We have integrated these discussions into the newly added sub-section "3.1 Validation of the simulation model" at the beginning of the "Results and discussion" section which located at page 8 line 190.

3. The FEM model description is incomplete, and important information is missing, including, software platform used, number of mesh elements, mesh independence study, convergence criteria, solver settings, and computational accuracy.

Response: We sincerely appreciate the reviewer for pointing out the incomplete description of the finite element model. The 3D model was established using the commercial software ANSYS which has now been specified in the revised manuscript page 8 line 186. The steady-state calculation used a fully coupled linear solver with an automatic nonlinear method. To guarantee solver stability, the initial damping factor was set to 1, the minimum damping factor to 1e-4, and an automatic recovered damping factor was enabled with a recovery factor of 0.75. The update step limit and step growth limit were set to 10 and 1, respectively. The convergence criteria were based on tolerance and residual termination techniques, with a maximum of 25 iterations, a tolerance factor of 1, and a residual factor of 1000. Regarding the mesh generation, the computational domain consists of 1,481,522 tetrahedral elements, 91,398 triangular elements, 2,321 edge elements, and 41 vertex elements. The average element quality reached 0.6953

Attachment Submitted filename: Response to Reviewers.docx

# Peer review: Decision Letter 1

**DOI:** 10.1371/journal.pone.0356685.r003

**Authors:** Azim Uddin

7 Aug 2026

Influence of semiconductive shielding protrusions on the electric field distribution in ±500 kV HVDC cables

PONE-D-26-17827R1

Dear Dr. Xu,

We’re pleased to inform you that your manuscript has been judged scientifically suitable for publication and will be formally accepted for publication once it meets all outstanding technical requirements.

Within one week, you’ll receive an e-mail detailing the required amendments. When these have been addressed, you’ll receive a formal acceptance letter and your manuscript will be scheduled for publication.

An invoice will be generated when your article is formally accepted. Please note, if your institution has a publishing partnership with PLOS and your article meets the relevant criteria, all or part of your publication costs will be covered. Please make sure your user information is up-to-date by logging into Editorial Manager at Editorial Manager® and clicking the ‘Update My Information' link at the top of the page. For questions related to billing, please contact billing support.

If your institution or institutions have a press office, please notify them about your upcoming paper to help maximize its impact. If they’ll be preparing press materials, please inform our press team as soon as possible -- no later than 48 hours after receiving the formal acceptance. Your manuscript will remain under strict press embargo until 2 pm Eastern Time on the date of publication. For more information, please contact onepress@plos.org.

Kind regards,

Azim Uddin, Ph.D.

Academic Editor

PLOS One

Additional Editor Comments (optional):

Reviewers' comments:

Reviewer's Responses to Questions

**Comments to the Author**

Reviewer #1: All comments have been addressed

Reviewer #2: All comments have been addressed

Reviewer #3: All comments have been addressed

**********

2. Is the manuscript technically sound, and do the data support the conclusions?

Reviewer #1: Yes

Reviewer #2: Yes

Reviewer #3: Yes

**********

3. Has the statistical analysis been performed appropriately and rigorously?

Reviewer #1: (No Response)

Reviewer #2: N/A

Reviewer #3: Yes

**********

4. Have the authors made all data underlying the findings in their manuscript fully available?

The PLOS Data policy

Reviewer #1: Yes

Reviewer #2: Yes

Reviewer #3: Yes

**********

5. Is the manuscript presented in an intelligible fashion and written in standard English?

Reviewer #1: Yes

Reviewer #2: Yes

Reviewer #3: Yes

**********

Reviewer #1: (No Response)

Reviewer #2: The authors have addressed the comments raised in the previous round of review and made the corresponding revisions. I have no further comments on the revised manuscript.

Reviewer #3: (No Response)

**********

what does this mean?). If published, this will include your full peer review and any attached files.

If you choose “no”, your identity will remain anonymous but your review may still be made public.

**Do you want your identity to be public for this peer review?**  For information about this choice, including consent withdrawal, please see our Privacy Policy

Reviewer #1: No

Reviewer #2: No

Reviewer #3: **Yes:**  Dr. Muhammad Wahab Hanif

**********

# Editor report: Acceptance letter

**DOI:** 10.1371/journal.pone.0356685.r004

**Authors:** Azim Uddin

PONE-D-26-17827R1

PLOS One

Dear Dr. Xu,

I'm pleased to inform you that your manuscript has been deemed suitable for publication in PLOS One. Congratulations! Your manuscript is now being handed over to our production team.

At this stage, our production department will prepare your paper for publication. This includes ensuring the following:

* All references, tables, and figures are properly cited

* All relevant supporting information is included in the manuscript submission,

* There are no issues that prevent the paper from being properly typeset

You will receive further instructions from the production team, including instructions on how to review your proof when it is ready. Please keep in mind that we are working through a large volume of accepted articles, so please give us a few days to review your paper and let you know the next and final steps.

Lastly, if your institution or institutions have a press office, please let them know about your upcoming paper now to help maximize its impact. If they'll be preparing press materials, please inform our press team within the next 48 hours. Your manuscript will remain under strict press embargo until 2 pm Eastern Time on the date of publication. For more information, please contact onepress@plos.org.

You will receive an invoice from PLOS for your publication fee after your manuscript has reached the completed accept phase. If you receive an email requesting payment before acceptance or for any other service, this may be a phishing scheme. Learn how to identify phishing emails and protect your accounts at https://explore.plos.org/phishing.

If we can help with anything else, please email us at customercare@plos.org.

Thank you for submitting your work to PLOS ONE and supporting open access.

Kind regards,

PLOS ONE Editorial Office Staff

on behalf of

Dr. Azim Uddin

Academic Editor

PLOS One
